# Traditional Chinese Medicine Modernization in Diagnosis and Treatment: Utilizing Artificial Intelligence and Nanotechnology

**DOI:** 10.1002/mco2.70596

**Published:** 2026-02-03

**Authors:** Wenqi Yu, Mengzhen Chen, Xueqi Tan, Xi Wei, Fan Sun, Hua Yan, Xue Xu, Hongcai Shang

**Affiliations:** ^1^ Department of Cardiology, Wuhan Asia Heart Hospital Affiliated to Wuhan University of Science and Technology Wuhan University of Science and Technology Wuhan China; ^2^ School of Medicine Wuhan University of Science and Technology Wuhan China; ^3^ College of Life Sciences and Health Wuhan University of Science and Technology Wuhan China; ^4^ Dongfang Hospital Beijing University of Chinese Medicine Beijing China

**Keywords:** artificial intelligence, four diagnostic methods, modernization, nanotechnology, traditional Chinese medicine

## Abstract

Traditional Chinese medicine (TCM), consisting of a complete TCM diagnosis and treatment system, is a valuable treasure in the long river of Chinese clinical history. However, the subjective diagnosis, ambiguous mechanisms, and complex formulas make it slightly lag behind the development of modern medicine. With the emergence of novel technologies such as artificial intelligence (AI) and nanotechnology, TCM modernization has regained its promise of hope. In this review, we provide an overview of applications of AI and nanotechnology to assist TCM modernization. Firstly, we summarize the auxiliary TCM diagnosis approaches based on machine learning and deep learning, which facilitate “four diagnostic methods” (inspection, auscultation–olfaction, inquiry, and pulse palpation) with standard and quantifiable data collection, and objective syndrome differentiation and diagnostic decisions. Secondly, a comprehensive overview of the nanotechnology used to enhance the therapeutic effects of TCM is provided, including optimizing TCM formulas and enhancing active targeting. Finally, we summarize the current challenges, clinical translation, and future perspectives of AI, TCM diagnosis, and nanotechnology. Our review and insights aim to provide valuable guidance for the continued advancement of TCM modernization.

## Graphical abstract

Traditional Chinese medicine (TCM) faces great challenges during the development of modern medicine. Utilizing novel technologies such as artificial intelligence (AI) and nanotechnology facilitates TCM with great potential in precision diagnosis and improved treatments efficacy.

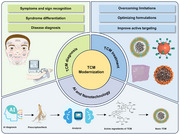

## 1 Introduction

Traditional Chinese medicine (TCM), as an essential component of China's ancient medical system, originated from ancient Chinese philosophy. Through centuries of clinical practice, it has developed into a unique and comprehensive medical system consisting of its own diagnostic and therapeutic methods independent of Western medicine. The four diagnostic methods are the basic basis of TCM diagnosis, which is composed of inspection, auscultation–olfaction, inquiry, and pulse palpation (IAOIP). The information obtained from the four diagnostic methods is integrated and comprehensively analyzed to understand the cause and mechanism of disease. Based on the diagnosed symptoms, the treatment of TCM is divided into external treatment (acupuncture, cupping, massage, ear acupoint, etc.) and internal treatment (herb, animal, mine medicine) [1]. Compared with Western medicine, which is based on physiopathology with specific targets, causes, and symptoms, TCM pays more attention to a holistic view to realize disease prevention and treatment by adjusting the internal balance of the human body [2]. The various components of the body are considered as a whole centered around the five Zang‐organs in TCM theory and are mutually restricted in terms of structure and function to achieve a normal physiological state of internal and external balance [3, 4]. Therefore, the holistic TCM theory has great prospects in regulating the systemic body function, which is especially promising under the background of personalized therapy and disease prevention [5].

However, TCM diagnosis relies heavily on the experience and intuition of physicians, lacking standardized and quantifiable methods, which leads to a high degree of subjectivity of diagnostic results [6, 7]. Additionally, TCM practitioners typically require years of study and practical experience to acquire sufficient theoretical knowledges and diagnostic skills [8]. Furthermore, due to the ambiguity and uncertainty of TCM theories and practices, TCM relies more on inheritance and long‐term clinical practice, significantly limiting its widespread application [9, 10]. For therapeutic treatment, TCM and their active ingredients face challenges such as unclear targets and mechanisms [11, 12], poor solubility [13, 14], low bioavailability [13, 15], short half‐life [16], and instability in biological milieu [14], which significantly impact their efficacy, bioavailability, and toxicity, thus limiting their broad applications in modern clinical treatment [17]. The clinical efficacy of nanomedicines is also an issue of concern, as only a few nanomedicines have been approved by the United States Food and Drug Administration (US FDA) in the past 30 years, and only 14% have shown significant clinical efficacy [18].

To address these challenges, the modernization of TCM has become an important direction for future development. Recent years, the development of big data and artificial intelligence (AI) have shown promising applications in TCM modernization, such as disease diagnosis, syndrome differentiation, targets identification, mechanism studies, combinational therapy, personalized medical therapy, and so on [19, 20, 21]. The four diagnostic methods, as the core diagnostic techniques of traditional TCM, have gained technological support and innovation from AI focusing on characteristic sign collection, syndrome differentiation, and disease diagnosis [22, 23, 24, 25, 26]. By introducing AI, TCM doctors can collect the symptom sign with standardization and quantification [27]. Moreover, objective conclusions of syndrome differentiation and diagnostic results can be given after being analyzed by AI, advancing to a precise and efficient diagnosis of modernized TCM [28].

Nanotechnology is another emerging technology that enables precise encapsulation, targeted delivery, and controlled release of the active ingredients of TCM. Over the past decade, nanoformulations of TCM, such as liposomes, polymer nanoparticles, micelles, and biomimetic nanocarriers, have demonstrated enhanced therapeutic effects, reduced systemic toxicity, and higher stability in preclinical and clinical studies, and have also been able to reveal the mechanism of action of complex TCM formulas [14]. Moreover, the introduction of AI has provided new solutions for nanomedicine research in terms of formula optimization, mechanism elucidation, and individualized treatment design. This collaborative integration not only enhances the understanding of the mechanism of TCM but also accelerates the modernization process of TCM and provides possibilities for its application worldwide.

**FIGURE 1:**
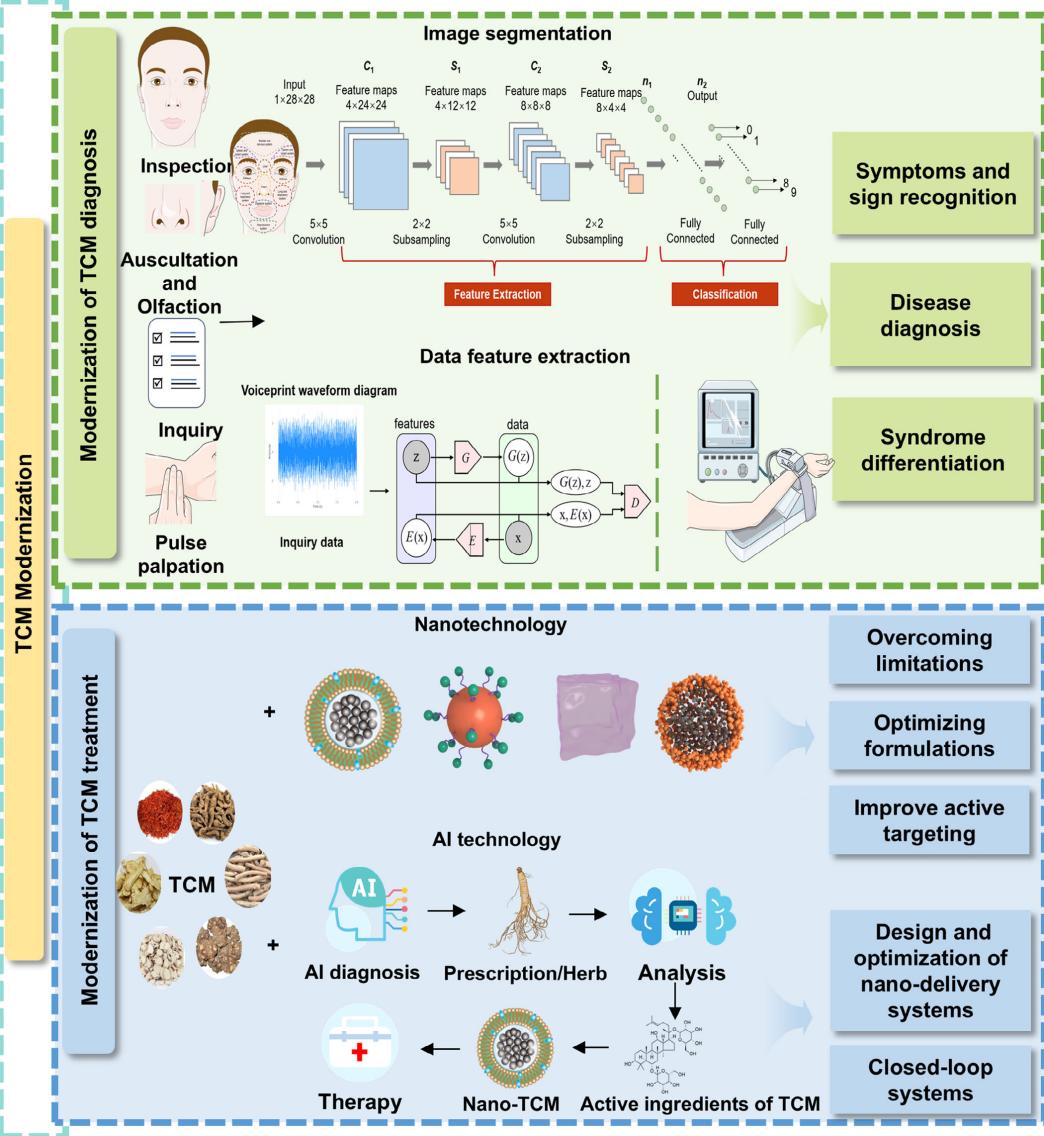
Modernization of TCM through AI and nanotechnology for improved diagnosis precision and treatment efficacy. AI‐assisted multimodal data analysis has been applied in symptoms and sign recognition, disease diagnosis, and syndrome differentiation. And nanotechnology together with AI further boosts the development of TCM therapy.

In this review, we summarized the recent progress of diagnosis and treatments during the modernization of TCM (Figure 1). First, we discussed the application of AI in the four diagnostic methods of TCM and its role in supporting syndrome differentiation and disease diagnosis. Second, we elaborated on the nanotechnology applied in the modernized TCM treatments. Then, the combination of AI, nanotechnology, and other novel technologies was discussed to reveal the mechanism of TCM formulas. Finally, we gave a comprehensive discussion of future challenges and outlooks during the process of TCM modernization.

## 2 AI‐Powered Data Mining for TCM Diagnosis and Mechanistic Insights

TCM diagnosis is grounded in the “four diagnostic methods” that collectively capture multidimensional physiological and pathological information of the human body [29]. These diagnostic approaches are highly dependent on practitioners’ experience and subjective judgment, leading to inconsistencies in clinical interpretation. In recent years, the integration of AI and data‐driven analytics has enabled the digitalization and quantification of these traditional diagnostic modalities. By converting qualitative signs such as tongue color, facial complexion, pulse waveform, and voice tone into measurable data, AI technologies facilitate objective assessment and pattern recognition [30]. Moreover, the development of AI facilitates integration of multiomics and systems pharmacology to unveil the complex mechanisms of TCM.

### 2.1 Digitalizing and Quantifying the TCM Four Diagnostic Methods

#### 2.1.1 Inspection

Inspection stands as the foremost of TCM four diagnostic methods. By observing a patient's facial expression, complexion, physique, posture, tongue appearance, and facial features, practitioners can preliminarily assess the body's Qi and blood vitality, as well as the functional state of internal organs, thereby inferring the nature and progression of disease. Since inspection relies heavily on visual diagnostic information and clinical experience, lacking uniform quantitative standards, interpretations often vary among practitioners. In recent years, some studies have attempted to introduce objective imaging documentation and image analysis methods to reduce subjective factors in visual diagnosis, making the relevant information more comparable and reproducible. This trend provides new technological support for traditional experiential diagnosis and lays the foundation for standardizing and modernizing visual diagnosis research. This AI‐assisted inspection facilitates the objectification and standardization of TCM in comparison with traditional techniques, which boosts the modernization of TCM [31].

**FIGURE 2:**
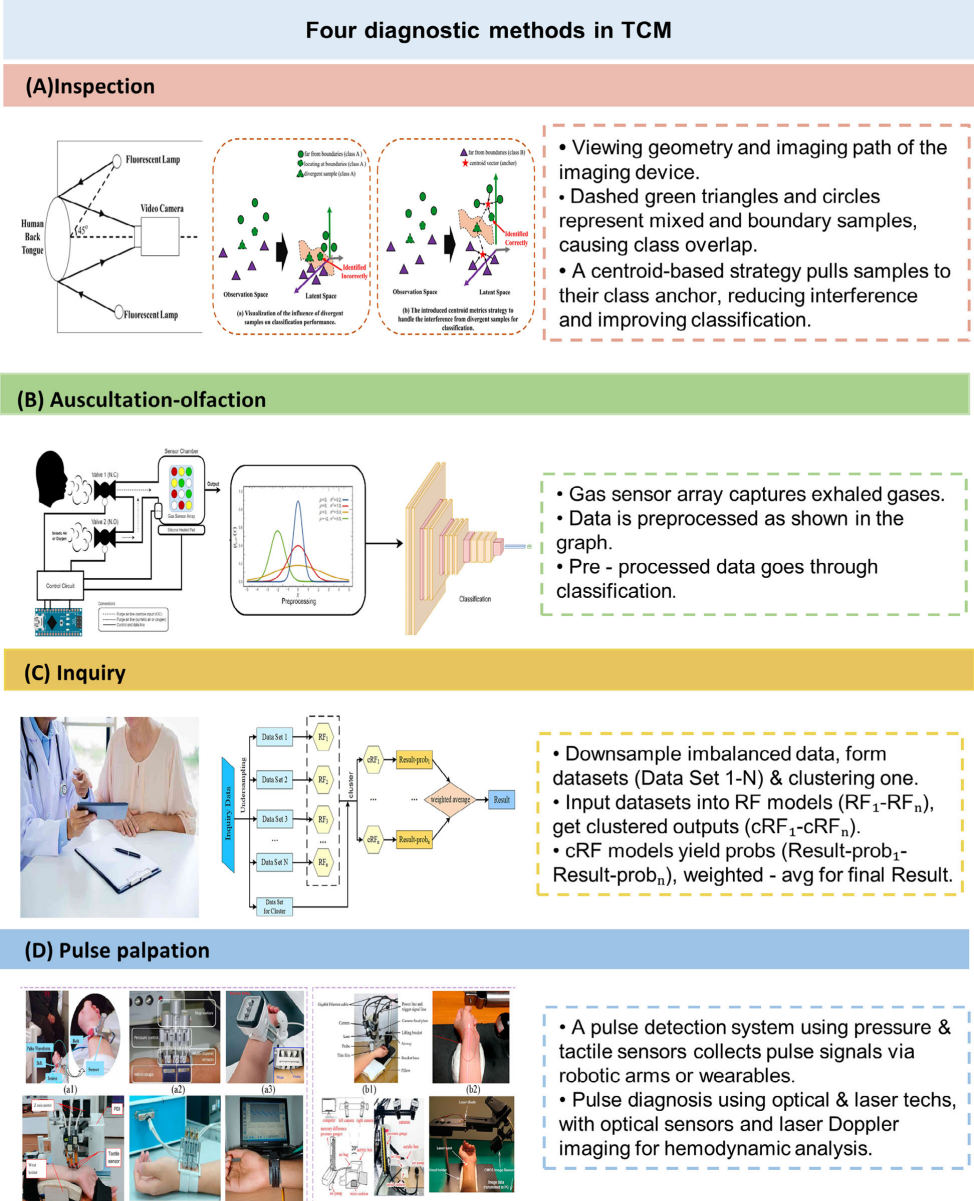
The diagram of TCM diagnosis by the four diagnostic methods: IAOIP. (A) Schematic diagram of the facial image acquisition device and the entire framework of the MFLCM method. Copyright 2024, Elsevier [33]. (B) The electronic nose collects odors and analyzes the data using machine learning. Copyright 2022, Elsevier [35]. (C) Architecture diagram of the consultation model constructed by Cluster‐RFs. Copyright 2022, Elsevier [36]. (D) Prototypes of contact and contactless pulse acquisition systems. Copyright 2022, Elsevier [37].

Facial diagnosis is a key part of TCM inspection, TCM doctors observe facial color, gloss, shape, and expression to infer the internal physiological and pathological status, which takes a lot of experience for diagnosis accuracy. Research in this area has primarily focused on facial image processing, facial feature recognition, and the facial diagnosis system establishment. Observation of the patient's facial color and radiance is an important method of clinical diagnosis in TCM. Li et al. used principal component analysis (PCA) and linear discriminant analysis (LDA) methods to design a face glossiness classification model, which classify the glossiness information of face images into red, green, blue, and gray. The prediction accuracy of LDA in the color space reaches up to 98%, providing an automatic quantitative method for TCM diagnosis based on face images. And the consistency of the diagnosis with the experts in clinical diagnosis is 81%, which has practical application value [32]. As for the facial image processing and dataset optimization, Zhang et al. proposed a novel multifeature learning approach named Multi‐Feature Learning with Centroid Matrix (MFLCM), which mitigates the impact of scattered samples on the accurate classification of samples at the boundary, such as those with different environmental factors, living conditions, or genetic factors. A discriminator integrating the centroid matrix strategy was introduced, capable of adapting to the classifier of the unified model, thereby enabling better extraction of facial skin features for disease diagnosis (Figure 2A) [33]. Additionally, Wen et al. created a system that integrated tongue and facial images for TCM diagnosis, utilizing AI technologies like image recognition and ML for comprehensive application of big data of TCM [34].

Tongue diagnosis includes observing changes of tongue body, coating, and sublingual veins for health condition assessment. TCM considers tongue as a mirror to reflect the physiological and pathological conditions of internal organs [38]. Traditional tongue diagnosis is affected by many factors, and its differential diagnosis has not been widely recognized [39]. Research in tongue diagnosis has focused on using deep learning (DL) to correct, segment, and recognize tongue images. Ma et al. adopted a system framework that used a deep convolutional neural network (CNN) to automatically recognize tongue structure from natural tongue images for tongue coating detection and correction, as well as tongue structure recognition. Based on this, they proposed a new complexity sensing classification method. The adverse effects of changes in environmental conditions and uneven distribution of tongue images on the performance of structure recognition are well treated. The clinical trial results on clinical tongue images showed that the method could effectively improve the recognition accuracy of health status. The tongue diagnostic instrument is the product of the modernization of tongue diagnosis, which has advantages in the standardization and objectivity in clinical practice. Zhang et al. proposed an automatic detection model for tooth marks and cracks on the surface of tongue based on improved YOLOv5, which added the SimAM—CSP module to the backbone, inserted a bottleneck attention module, and adjusted the feature fusion structure. Using EIoU instead of GIoU speeded up training and improved accuracy, the improved model exhibited an mAP of 79.5%, 6.3% higher than the original one [40]. ResNet34, an AI network framework based on CNN, was applied to this dataset to automatically extract image features and realize tongue image classification. It is verified that ResNet34 architecture can better complete the task of tooth mark and tongue texture feature recognition. The model architecture displays excellent performance and strong generalization ability, which is more accurate to analyze the user's health status [41].

Eye diagnosis is a method to diagnose diseases by observing the changes of the spirit, color, shape, and appearance of various parts of the eye. According to TCM, the eye is the epitomized of the five Zang‐organs, which can reflect the diseases of Zang‐organs, six meridians, seasonal pathogens, and epidemic diseases [42]. Traditional eye diagnosis is limited to the visual observation of some morphological features outside the eye, while modern machine vision technology can detect lesions in the fundus location. Current research of eye diagnosis focuses on using ML or DL to process fundus images and achieve objective diagnosis. Fundus multispectral imaging (MSI) provides a series of narrow‐band images to visualize different depths of the retina and choroid. Sui et al. proposed a weakly supervised MSI image registration network (MSI‐R‐Net), which used vascular segmentation labels to provide spatial correspondence for multispectral fundus image registration, and a feature balance module was used to connect the aggregation layers better. Moreover, a multiresolution automatic context structure was proposed to adapt to the registration task [43]. The trained model can predict pixel spatial correspondence without labeled vessel information, with high registration accuracy. Retinal fundus imaging is an important diagnostic tool in ophthalmology for early detection and monitoring of various ocular diseases. Zhao et al. proposed a generative adversarial network composed of a generator and a discriminator, which could effectively improve the perceived quality of retinal fundus images. An artifact loss function was introduced to enhance the generative adversarial network, so that the difference between high‐resolution images and restored images could be measured more accurately. The experiment results showed that the generative adversarial network‐based novel technology exhibited better perceived quality compared with the existing super‐resolution imaging technology [44].

Hand diagnosis is a method to diagnose diseases by observing the changes of palm print, palm color, palm shape and nail. TCM considers that the hand connects the Zang‐organs through the meridians of the human body. As the heart powers the blood flow and pumps it to five Zang‐organs it will be reflected in the color and vein of the hands once the heart is impaired [45]. Research in AI‐assisted hand diagnosis is limited, the existing studies mainly focus on exploring the relationship between hand morphology and disease diagnosis [46, 47]. Liu et al. confirmed the correspondence between specific positions of the hand and the viscera by basic experiments [48, 49]. Liu et al. summarized the hand diagnosis method through hand color and hand shape based on their clinical practice experience. By collecting and analyzing hand images, the sensitivity of the hand diagnosis method based on Qi, color, and morphology in diagnosing carotid atherosclerotic lesions is 82.2%, and the specificity is 63.3%. The sensitivity in diagnosing coronary artery disease is 80.2%, and the specificity is 68.7% [50].

#### 2.1.2 Auscultation–Olfaction

TCM theory posits that a person's voice and scent reflect the physiological functions of their organs and the balance among Qi, blood, and body fluids. Auscultation–olfaction in TCM includes both listening to sounds and smelling odors, which help the practitioner understand the physiological and pathological changes in the internal organs [51]. Traditional auscultation–olfaction relies heavily on the practitioner's experience, making it difficult to detect subtle changes in sounds and smells. By capturing and analyzing sound waves and chromatographic data, AI provides considerable assistance for objective and accurate diagnosis.

Auscultation is based on the patient's vocalization, language, breathing, coughing, sneezing, vomiting, hiccups, belching, bowel ringing, and other sounds to determine whether there are abnormalities. Research in AI‐assisted auscultation focuses on analyzing voice signals. The first step in the modernization of TCM auscultation is to identify and analyze the voice for quantitative analysis. Yang et al. innovatively proposed four acoustic parameters: average zero crossing times, local peak and valley changes, the first and second peak frequency changes, and spectral energy ratio [52], which provided a feasible way and exhibited great potential to classify patients between Qi, Yin, and Yang. Zhang et al. proposed an ensemble convolutional bidirectional long short‐term memory (BiLSTM) network with optimal parameter selection for sound classification, showing advantages in identifying effective network configurations and generating temporal dynamics to improve sound classification performance [53]. Luo et al. proposed an adaptive scale audio feature extraction method, which is used to calculate window sizes and hop lengths at different scales to generate a multiscale Mel‐spectrogram (MSMel‐spectrogram). A CNN model with a channel attention mechanism was proposed to extract the features of the MSMel‐spectrogram. The channel attention mechanism can capture the channel information, thus improving the model performance. The average accuracy of classifying whooping cough in the cough sound dataset is 90.5% [54].

Olfactory diagnosis is a method to detect diseases by detecting the odors of a patient's body, secretions, excretions, and the ward. Currently, research on AI‐assisted olfaction mainly focuses on aspects such as sampling methods, enrichment techniques, data processing, and the construction of disease prediction models [55]. The ReCIVA alveolar gas sampling device launched by Olwstone Medical can simultaneously capture samples of both whole respiration and alveolar respiration [56]. Zhong et al. introduced the main enrichment methods and materials for exhaled breath. After exhaled breath is collected by gas bags, stainless steel containers, adsorption traps, and so on, it is usually preconcentrated and enriched by solid‐phase microextraction or thermal desorption, and then analyzed by methods such as gas chromatography mass spectrum, and ion mobility spectroscopy. Data processing includes data preprocessing, which involves denoising, baseline correction, and so on, feature extraction, as well as modeling and classification. Techniques such as PCA, LDA, and orthogonal partial least squares discriminant analysis (OPLS‐DA) are utilized to extract representative features from the original data. This process reduces the data dimension and enhances the accuracy of subsequent classification or quantitative analysis. Using algorithms such as the K‐nearest neighbor (KNN) in ML, the support vector machine (SVM), the artificial neural network (ANN), and the CNN, recurrent neural network in DL, an association model is established between exhaled breath data and specific diseases or physiological states [57]. Cries Avian and others used an electronic nose device to collect breath samples, and processed the collected gas signals through two types of constructed models to achieve the classification of the subjects (Figure 2B) [35]. One used a 1D convolutional layer, and the classification consisted of four processing blocks: sensor, preprocessed signal, feature extraction, and classification. The gas sensor produced a signal of 4000 samples × 8 channels (numbered gas sensors). Then the signal was processed using signal preprocessing (standard scaler). The other model used a 2D convolutional layer and was able to directly utilize a CNN to extract and classify data simultaneously.

#### 2.1.3 Inquiry

Inquiry in TCM is a method of diagnosing diseases by systematically asking the patient or their companions to understand the beginning, development, and treatment history of the disease; current symptoms and other relevant information are also included. This method helps gather comprehensive data that other diagnostic methods might miss [58]. Traditional TCM inquiry often involves complex, interwoven information with high data dimensionality, placing high demands on the practitioner's knowledge and the patient's ability to articulate their symptoms. Moreover, TCM case samples are characterized by nonstandard term expression, complex data attribute, and complex structure. Yang et al. proposed the Cluster‐RFs ensemble diagnostic model to address the issues of missing features and class imbalance in TCM inquiry scales. This method integrated the Bagging strategy with decision trees as base classifiers, dividing majority class samples into subsets combined with minority class samples to ensure class balance. K‐means clustering was introduced, utilizing prediction probabilities from random forests on the test set to optimize base classifier selection, maximizing cluster diversity and enhancing model generalization. Moreover, weighted averaging was applied to improve diagnostic performance (Figure 2C) [36]. With the rapid development of AI, inquiry models can be standardized, which facilitates information collection with accuracy. He et al. developed a TCM inquiry‐assisted diagnosis algorithm that integrated bidirectional encoder representation from transformers (BERT) and graph convolutional network (GCN), utilizing digitized TCM literature to train a BERT natural language processing model that generates TCM term vectors. These vectors, combined with symptoms, severity, and other indicators, serve as input features for a GCN model. Compared with other models, the BT‐GCN model achieved a higher accuracy rate of 97.6% on test samples [59]. TCM believes that the human body is a complete organism, and the Zang‐organs are interrelated, so it is far from enough to stay in the study of a specific system of the Zang‐organs. Li et al. adopted the symptom questioning model based on the frequent pattern mining algorithm in association analysis. They used the cross‐merging method to establish the TCM symptom inquiry strategy of single‐system symptom questioning and multisystem comprehensive symptom questioning. The purpose of this was to obtain the key condition information of patients in the shortest time and with the highest efficiency. And it also realized the breakthrough from single‐system inquiry to five‐system comprehensive inquiry. Through the two symptom questioning modes of single system and five system, the condition information of patients can be obtained efficiently [60].

#### 2.1.4 Pulse Palpation

Pulse palpation is when the physician uses their hands to touch, press, and feel specific parts of the patient's body to assess their health status and diagnose diseases. In TCM, pulse diagnosis is narrowly defined as palpation, which interprets the abnormal pulsation of superficial arteries to analyze the state of the internal organs [61]. At present, there is a problem of “the heart is clear, the finger is difficult to understand.” In clinical diagnosis, the accurate identification of pulse characteristics requires doctors to have rich clinical experience, and the diagnostic results are largely affected by the subjective consciousness of doctors. Therefore, it is of great significance to take advantage of AI to realize the objectification and intelligence of pulse diagnosis. At present, the research of intelligent pulse diagnosis mainly focuses on using sensors to objectify pulse in the form of pulse graphics and using algorithms to diagnose diseases according to pulse signals. There are already two types of pulse acquisition systems, namely, contact and noncontact ones, which can obtain the time series of parameters that change with pulsations at three positions under different pressing pressures (Figure 2D) [37]. Zhang proposed a graph‐based multichannel feature fusion method that used pressure and photoplethysmography sensors to capture three channels of wrist pulse signals, effectively utilizing multichannel features for wrist diagnosis [62]. Pulse diagnosis in TCM includes 28 pulse signs, and some of which might occur simultaneously. “Four pulse diagnosis” was introduced by Shaowu Liu who created a new theory of TCM including the “three parts six diseases theory.” The “Four pulse diagnosis” simplifies the complex pulse signs, thereby reducing the difficulty of standardization. Based on this theory, Shen et al. constructed a multilabel pulse state classification model with an accuracy of 92.74%, which integrated voice analysis with pulse conditions. The method provided a viable strategy to obtain pulse conditions without specialized equipment [63]. Continuous monitoring of pulse waves plays an important role in reflecting physical condition and disease diagnosis for long‐term observation. Kang et al. proposed a new wearable real‐time pulse wave monitoring system based on a new flexible composite sensor. The flexible composite sensor was composed of custom‐packaged pressure sensor, signal stabilization structure, and micropressurization system to complete the stable acquisition of pulse wave signal under continuous variable pressure, and the real‐time algorithm completed the analysis of pulse wave peak trend. The optimal pulse wave can be quickly and accurately located for different individuals with an accuracy of 95% [64].

### 2.2 | **From Data to Diagnosis: AI for Disease and Syndrome Pattern Recognition**

With the advancement of algorithms and instruments, the AI‐assisted integration of the four diagnostic methods enhances the sign collection and analysis of IAOIP, which facilitates the TCM diagnosis with objectivity [65]. The application of AI in assisting disease and syndrome differentiation facilitates a more comprehensive understanding of complex syndromes, contributing to more accurate and personalized treatment strategies.

#### 2.2.1 Disease Diagnosis

Disease diagnosis is one of the main application scenarios integrating AI and TCM. Currently, most studies focus on utilizing traditional ML and DL algorithms to assist TCM diagnosis. In TCM, each of the four diagnostic methods has its own emphasis, and numerous studies have demonstrated the accuracy of using one of these methods for disease diagnosis. As TCM emphasizes a holistic view, and ML, through algorithms, also needs to learn from more diverse data to analyze and predict new datasets, there are emerging research of combining the four diagnostic methods for a more comprehensive disease diagnosis. As shown in Table 1, the AI‐assisted IAOIP is being applied revolutionarily in variable diseases with great potential.

**TABLE 1:** Summary of the application of AI‐assisted IAOIP in disease diagnosis.

Diagnostic methods	Collected data	Technical methods	Diagnosed disease	Accuracy rate (%)	AUC	References
Facial diagnosis	Facial textures	support vector machine (SVM)	Diabetes mellitus	99.02	/	[66]
	Facial images	SVM	Endocrine metabolic syndromes	82.7‐92.0	/	[67]
		PCA k‐Nearest Neighbor (PCA‐KNN)		76.6‐89.0	/	
		AdaBoost		81.8‐93.5	/	
Tongue diagnosis	Tongue surface, tongue color	ResNet50	Precancerous lesions of gastric cancer	/	0.752	[38]
		multifeature learning method (MFL)	Diabetes mellitus	93.38	/	[68]
Eye diagnosis	Retinal image	Deep Learning (DL)	Coronary artery calcium	/	0.742	[69]
	RNFLT	SVM	Glaucoma	/	0.860	[70]
Auscultation–olfaction	Chemical composition of odor molecules	XGBoost	Lung cancer	79.31	/	[71]
			Chronic obstructive pulmonary disease	76.67	/	
Palpation diagnosis	Pulse waveform	Iterative sliding window (ISW)	Lung cancer	78.13	/	[72]
Inquiry, palpation diagnosis	Text, pulse signal	CNN‐BiLSTM, RFS	H‐type hypertension	87.78	/	[36]
IAOIP integrated diagnosis	Image of tongue, face, sublingual vein, pulse waveform, chemical composition of odor molecules	Shared autoencoder gaussian process (SAGP)	Diabetes mellitus	94.4	/	[73]

##### 2.2.1.1 Intelligent TCM in Inspection Diagnosis

Information obtained from inspection is quite rich, including local facial expressions, tongue, eyes, palms, and overall mental and physical conditions. When combined with AI, this can be applied to diagnose various diseases. Facial diagnosis has long been an important component of TCM, where doctors can diagnose diseases based on some typical facial features. Many endocrine and metabolic syndromes are associated with specific facial features, including wide‐eyed hyperopia, triangular face, saddle nose, low ears, microcephaly, dental hypoplasia, cleft lip, cleft palate, prominent eyes, and dermatologic manifestations [74]. At present, diabetes can be detected by noninvasive methods. Shu et al. used eight texture extractors to extract texture features in specific regions of the face. The experimental results showed that the best texture feature extraction method of SVM for diabetes detection is image gray level histogram and the detection accuracy is 99.02% [66]. Wu et al. established a facial image database of multiple endocrine metabolic syndromes and healthy controls. They quantified facial complexity for each syndrome by calculating the disease facial recognition intensity and trained an AI‐based facial recognition (AI‐FR) system using SVM, PCA‐KNN, and Adaptive Boosting (AdaBoost). The SVM diagnostic accuracy of the AI‐FR model ranged from 0.827 to 0.920, PCA‐KNN from 0.766 to 0.890, and AdaBoost from 0.818 to 0.935 [67]. Transdermal optical imaging can measure hemoglobin concentration changes in raw digital camera images representing facial blood flow fluctuations, which allows continuous monitoring of blood pressure using multilayer perceptron algorithms. Based on the above techniques, pulse signal extraction from facial features can provide a simple and convenient method for blood pressure measurement. Xing and colleagues proposed an AI framework using deep CNN to predict blood pressure [75]. They extracted pulse wave signals from 652 facial videos and compared the training results of nine models, finding that L‐VGG was the best prediction model with an overall accuracy of 90% on the dataset. The tongue is anatomically connected to the digestive system organs. Some studies have found that the characteristics of tongue surface and color can be used as indicators to assist the diagnosis of gastric cancer. Screening for precancerous lesions of gastric cancer (PLGC) is an important means of preventing gastric cancer. Ma et al. first constructed a DL model (Aitongue) based on tongue images for PLGC screening. A fivefold cross‐validation analysis of 1995 patients in an independent cohort showed that the Aitongue model had an AUC of 0.752 for screening PLGC [38]. Tongue diagnosis has also been proven to be an effective method for diagnosing diabetes mellitus (DM). Zhang et al. designed a general multifeature learning method (MFL) [68]. They represented color by the cluster center (color centroid point) of color points sampled from tongue images, and extended the descriptor to three color spaces, namely RGB, HSV, and LAB, to mine rich color information and utilize the complementary information among these three spaces. To make full use of the similar and complementary information, these two parts were jointly transformed into their respective label vectors, and the discriminative prior was effectively embedded into the model. The average accuracy of the proposed process for diagnosing DM using posterior tongue images is as high as 93.38%. Some researchers believe that eye diagnosis has significant value for screening, diagnosing, and predicting cardiovascular diseases [76]. Researchers used 216,152 retinal photographs from South Korea, Singapore, and the UK to train and validate a DL‐based algorithm (RetiCAC) for predicting the probability of coronary artery calcium (CAC). The results showed that RetiCAC outperformed single clinical parameter models in predicting the presence of CAC, with an area under the receiver operating characteristic curve of 0.742. It could effectively predict the risk of cardiovascular events in different cohorts [69]. Eye images can also directly diagnose eye diseases. Xu et al. established a new local image‐based DL method to automatically predict the peripapillary retinal nerve fiber layer thickness (RNFLT) by optical coherence tomography fundus photography [70].

##### 2.2.1.2 Intelligent TCM in Auscultation–Olfaction Diagnosis

AI‐assisted auscultation and olfaction for disease diagnosis and prediction usually first extract parameters that represent the essential characteristics of sound and odor signals and then use these parameters to identify pathological sounds and odors. TCM believes that voice changes occur in patients with lung disease. Song et al. studied the differences in voice characteristics between patients with pulmonary nodules and healthy individuals, finding that the voices of patients with pulmonary nodules were lower and weaker, with the size of the nodules affecting voice peaks. Additionally, patients with pulmonary nodules accompanied by hyperlipidemia exhibited more significant voice damage [77]. A meta‐analysis found that total talk time and speech rate, mean and variability of fundamental frequencies were significantly lower in patients with schizophrenia than in healthy people, while mean pause times were higher [78]. Zhao et al. used Praat software to analyze and extract acoustic features of recordings, such as jitter, shimmer, and pitch. They analyzed the acoustic differences between the two groups of subjects and the relationship between acoustic features and clinical symptoms in the patient group [79]. The study found differences in emotional expression in the voices of schizophrenia patients compared with healthy controls, providing an accurate and feasible method for assessing negative symptoms in schizophrenia.

Electronic nose technology is the most commonly used method for collecting and analyzing odors. Many studies have discovered relationships between odors and diseases using electronic nose technology, which can be applied to the development of intelligent olfactory diagnosis systems in TCM. One study used an electronic nose consisting of 10 MOS gas sensors to diagnose ovarian cancer (OC). The sensitivity and specificity of the KNN classification model for distinguishing OC patients from controls were 98 and 95%, respectively [80]. Researchers developed an electronic nose device based on a chemical gas sensor array with a ML algorithm. The XGBoost ensemble learning method achieved accuracies of 79.31 and 76.67% for classifying lung cancer and chronic obstructive pulmonary disease [71]. Some scholars have developed a TCM olfactory diagnosis system based on exhaled breath detection using a combination of gas chromatography–surface acoustic wave sensors. They established a model of the relationship between exhaled breath and spleen–stomach abnormalities using ANN, with an AUC of 0.930. The accuracy of identifying syndromes such as spleen Qi deficiency, spleen–stomach damp‐heat, and spleen–stomach cold deficiency all exceeded 84%, indicating its potential use in distinguishing spleen–stomach syndromes in TCM [81].

##### 2.2.1.3 Intelligent TCM in Inquiry Diagnosis

The modernization of inquiry diagnosis is hampered by the diversity of unstandardized descriptions. Also, the inquiry diagnosis usually needs to be considered in combination with other diagnostic methods for disease diagnosis, which makes it difficult for modernization and standardization. In recent years, with the rapid development of AI technology and other diagnostic methods, the modernization of inquiry diagnosis has gradually made some progress [82]. In TCM, the diagnosis and treatment of coronary heart disease (CHD) has a long history and rich experience, but the nonstandard interrogation process affects the diagnosis and treatment of TCM to a certain extent. Liu et al. designed a standardized TCM inquiry diagnosis scale for CHD and used multilabel learning (MLL) technology to build an inquiry diagnosis model based on the collected data. In this study, a popular MLL algorithm (ML‐kNN) was compared with two other MLL algorithms (RankSVM and BPMLL). Furthermore, a commonly used single learning algorithm kNN was used to further explore the effect of symptom selection on the diagnostic model. A total of 555 cases were collected to establish the CHD query model. ML‐kNN, RankSVM, BPMLL, and kNN models were constructed by fusing examination, pulse sensation, palpation and standardized inquiry information, and the average diagnostic accuracy was 77, 71, 75, and 74%, respectively [83]. In recent years, hypertension has become increasingly common with a high incidence rate, with up to 75% of patients reported to have H‐type hypertension. Yang et al. proposed a model that combined pulse diagnosis with inquiry diagnosis based on CNN‐BiLSTM. This model includes a pulse diagnosis model based on CNN‐BiLSTM and an inquiry diagnosis model based on ensemble clustering RFS. They used a grid search method to dynamically search for the optimal weights of the heterogeneous comprehensive model for classifying H‐type hypertension, which outperformed other typical ML models [36].

##### 2.2.1.4 Intelligent TCM in Palpation Diagnosis

Pulse palpation is one of the most important diagnostic indicators in TCM, allowing for noninvasive and convenient diagnosis by sensing the patient's pulse. The Jin's pulse diagnosis (JPD) method has recently gained recognition and validation in the medical field as a highly efficient new development. Zhang and colleagues, inspired by the JPD theory, proposed a novel signal analysis method for lung cancer detection. They developed an iterative sliding window (ISW) algorithm to segment denoised signals into individual cycles, analyzed the pulse waveform characteristics, and summarized 26 features. Using a cubic SVM, they classified the pulse waveforms of healthy individuals and lung cancer patients, achieving an accuracy of 78.13% [72]. Peripheral artery disease (PAD) alters the propagation and reflection characteristics of pulse waves in the arteries. Sina and colleagues developed a DL algorithm that diagnoses PAD by analyzing the pulse volume recording waveforms of the brachial and tibial arteries. The experimental results showed that its performance in detecting PAD was comparable to that of arterial blood pressure measurements [84]. Diseases in different organs or tissues can reveal uneven pulse waves at corresponding depths of the radial artery. Cui and colleagues proposed a pulse diagnosis method based on acoustic waveforms, dividing the wrist pulse vertically into five layers for the first time. When comparing the five‐layer acoustic waves of patients with stable CHD and relatively healthy individuals, they found that the radial artery in patients with stable CHD exhibited abnormal acoustic pulse waves [85].

##### 2.2.1.5 Intelligent TCM in Integration of the Four Diagnostic Methods

The four diagnostic methods constitute an integrated system in TCM. Through these methods, a TCM practitioner comprehensively perceives a patient's condition using their senses of sight, hearing, smell, and touch. By combining these diagnostic methods and leveraging AI to collect, integrate, and analyze patient information, the accuracy of disease diagnosis can be significantly enhanced. Li et al. proposed a novel multimodal learning approach for DM. This method captured raw images or signals from the tongue, face, sublingual region, pulse, and scent. Relevant features were then extracted, and a ML algorithm was developed to optimize the proposed model. In experiments conducted on a dataset comprising 548 healthy samples and 356 DM samples, the method demonstrated its superiority. In the multimodal classification method, the classification accuracy of shared autoencoder gaussian process (SAGP) latent variable model was 94.4% [73]. In TCM, fatigue is considered to be related to a decline in the whole or local functional state of the human body, a manifestation of Qi deficiency. Shi et al. developed an early detection method for fatigue by collecting tongue and pulse data. They used four ML models in classification experiments to differentiate between disease‐related fatigue and nondisease‐related fatigue. The results indicated that the classification performance of the combined “tongue‐pulse” data was superior to that of either tongue or pulse data alone, making it a useful tool for distinguishing between the two types of fatigue [86]. Polycystic ovary syndrome (PCOS) is one of the most common endocrine and metabolic diseases in women of reproductive age. Some researchers found that women showed differences in tongue color during the menstrual cycle, which can be used to evaluate women's health [87]. Wang et al. utilized ML techniques, focusing on tongue and pulse data, and found that a SVM model incorporating both factors could accurately predict the presence of PCOS [88]. Identification of constitution is a key link in the practice of TCM. Accurate diagnosis of constitution helps doctors to choose the most effective treatment method, which is not only suitable for patients, but also helps healthy people to better understand their physical condition and prevent diseases. Huang et al. extracted tongue image, voice, and pulse waveforms from inspection, auscultation, and pulse diagnosis, and the constitution scale questionnaire was further used to measure the constitution score. Pearson correlation analysis was used to analyze the correlation between each index and constitution score. Indicators related to the abnormal constitution score were identified, and those with higher correlation coefficients had greater relative weight in determining the corresponding components [89].

#### 2.2.2 Syndrome Differentiation

Syndrome represents a pathological summary of a certain stage of a disease's development within the body. Syndrome differentiation involves analyzing the data, symptoms, and signs collected through the four diagnostic methods to identify the disease's cause, nature, location, and the relationship between pathogenic and healthy factors [90]. Every disease has a certain natural process of development from onset, early stage, middle stage, and late stage to recovery, relapse, or deterioration. Moreover, different people might exhibit different syndromes even at the same stage of diseases, which is consistent with the classical TCM theory “one disease has different syndromes.” Therefore, syndrome differentiation plays a key role in therapeutic guidance. Based on the basic theory of TCM, including Yin and Yang, five elements, Zang‐organs, and meridians, several syndrome differentiation standards are proposed such as eight‐principle syndrome differentiation, Zang‐organs syndrome differentiation and six‐channel syndrome differentiation. Through these syndrome differentiation methods, the basic theory of TCM is connected with syndromes. Therefore, syndrome differentiation is the key link of the combination of basic theory and clinical practice [91].

According to the classification and determination of TCM constitutions, objective indicators such as tongue and pulse features serve as important reflections of the balance of Qi and blood, Yin and Yang, and the functional state of the internal organs, thereby assisting in constitution identification and preventive treatment (“treating diseases before their onset”). For instance, a light‐red tongue with tooth marks and a moderate pulse is characteristic of a balanced constitution, whereas a pale, tender tongue with a deep and slow pulse is often associated with Yang deficiency. These traditional diagnostic features provide direct visual evidence for syndrome and constitution differentiation in TCM. [92] With the development of AI in TCM syndrome research, scholars have sought to digitize and quantify these diagnostic indicators to achieve objective and standardized syndrome identification. Wen et al. developed a zero‐shot learning model for tongue constitution recognition, which integrates DL‐extracted visual features such as tongue color, morphology, and coating with TCM diagnostic categories. The model establishes a semantic mapping between AI features and traditional syndromes—for instance, identifying pale red, tender, and scalloped tongues as Qi deficiency, and red tongues with yellow‐greasy coating as Damp‐Heat syndrome—thereby enhancing the interpretability of AI‐assisted syndrome differentiation [93]. Research indicates that TCM constitutions exhibit characteristic patterns in pulse wave parameters: Qi deficient and cold constitutions are positively correlated with the augmentation index, Yang‐deficient individuals show lower dP/dt max, while damp‐heat constitutions demonstrate higher subendocardial viability index. These findings translate theoretical TCM concepts such as “Qi deficiency leading to blood stasis” and “Yang deficiency causing cold congelation” into quantifiable vascular functional indicators, establishing a bridge between constitution identification and modern physiological mechanisms [94].

CHD is one of the most common cardiovascular diseases, and TCM has exhibited unique advantages in the treatment of CHD with good curative effects. Previous studies have shown that Qi deficiency syndrome and phlegm stasis syndrome are the basic syndromes of patients with CHD [95]. Based on this, Ren et al. used a TCM intelligent diagnostic instrument to collect tongue, facial, and pulse information from participants. Then, they applied a logistic regression model to construct a diagnostic model for phlegm and blood stasis syndrome in CHD, which achieved an AUC of 0.825 [89]. Rheumatoid arthritis (RA) is a systemic inflammatory autoimmune disease, which can be divided into damp‐heat obstruction syndrome, phlegm and blood stasis obstruction syndrome, liver and kidney deficiency syndrome, and wind and cold obstruction syndrome. Therefore, selecting appropriate features and improving the performance of syndrome classification model is the basis for future treatment. Xie et al. applied five feature selection rules and six models to classify TCM syndromes related to RA. The classification accuracy with feature selection was higher than that with all features, achieving a maximum accuracy of 0.88 with ANN [96]. From a ML perspective, TCM dialectics can be viewed as a complex model whose input is the four diagnostic information of patient and the output is the syndrome type. Huang et al. developed a multidimensional, high‐sparsity, multiclassification algorithm model suitable for TCM syndrome differentiation. This model standardized relevant terms in EMRs according to TCM symptoms and evidence‐based criteria, which further classified different symptoms and signs based on the four diagnostic methods in TCM to structure the case data. The established model achieved a classification accuracy of 96.21% for different syndrome types in 5273 real cases of dysmenorrhea [97]. The definition of rare diseases in TCM mainly refers to diseases with low incidence, which is difficult to obtain enough TCM clinical records, posing challenges to current AI models. Li et al. proposed a dual enhancement method based on transfer learning (TLDA) that enhanced the limited EMR data at both the sample and feature levels, enriching the pathological and medical information during the training process. This approach assisted doctors in diagnosing rare diseases accurately based on patients’ target symptoms and medical histories. TLDA is not only suitable for TCM, but can also be extended to Western medicine disease diagnosis scenarios if a migrated medical language model or corpus is available [98]. To improve the transparency and reproducibility of AI‐assisted diagnosis studies in TCM, we summarized the basic information of datasets used in representative research (Table 2).

**TABLE 2:** Dataset characteristics of AI‐assisted diagnostic studies based on TCM‐related indicators.

Collected data	Data source	Sample size	Age range	Study population (patients vs. controls)	Disease	Remarks	References
Facial images	Public database	2772	/	462/2310	Endocrine and metabolic syndromes	The disease group and the control group were randomly selected at the ratio of 5:1 matched by sex, age, and race.	[67]
Sublingual vein image	Guang Dong Provincial TCM Hospital	889	/	439/450	Diabetes mellitus	/	[68]
Acoustic features	Beijing Huilongguan Hospital	158	16–30	79/79	Schizophrenia	/	[79]
Breath samples	Public database	34	>18	20/14	Chronic obstructive pulmonary disease	High age parameter's standard deviation	[35]
Pulse acoustic waveforms	Shandong Provincial Famous TCM Inheritance	90	42–78	45/45	Coronary heart disease	/	[85]

#### 2.2.3 Model Interpretability and Explainable Outputs

In the application of AI to the four diagnostic methods of TCM, model interpretability is a crucial factor for achieving clinical trust and integration with traditional theory. For example, Ma et al. developed a DL model based on AiTongue for PLGC. During prediction, the model is able to highlight key regions of the tongue, and interpretability analysis revealed that thick coating, yellow coating, dark tongue body, fissures, and greasy coating contributed most to classification. These tongue features are highly consistent with traditional TCM tongue diagnosis principles [38]. The study by Song et al. on the phonetic characteristics of patients with pulmonary nodules showed that AI or statistical analysis models primarily relied on the pitch, loudness, and voice peak variations to distinguish patients from healthy individuals. Nodule size and the presence of hyperlipidemia contributed most to these voice features. By linking the model outputs to specific phonetic characteristics, clinicians can intuitively understand the basis of the model's decisions. This interpretability analysis aligns well with the TCM theory that lung diseases are associated with low or weak voice [77]. Zhang et al. extracted 26 key pulse waveform features using an ISW algorithm and employed a SVM to classify healthy individuals and lung cancer patients. Interpretability analysis indicated that pulse peak height, waveform amplitude, and rising/falling slope were the most influential features for classification. These features correspond closely to traditional TCM pulse diagnosis indicators: pulse peak height corresponds to pulse strength (the forcefulness of the pulse), waveform amplitude corresponds to pulse shape (width, depth, and fullness), and rising/falling slope corresponds to pulse rhythm (rate, regularity, and smoothness) [72].

### 2.3 Beyond Diagnosis: AI in Unraveling TCM Mechanisms via Systems Pharmacology and Multiomics Integration

Understanding the mechanism of TCM is a crucial part of TCM modernization, which provides theoretical basis based on modern medicine instead of practitioners’ experiences. However, the characteristics of multisyndromes, multicomponents, multitargets, and multipathways make it difficult for TCM mechanism analysis, leading to limited progress so far. With the development of AI, its ability to process a variety of large amounts of data exhibits the potential for TCM mechanism analysis, such as integrating systems pharmacology and multiomics data. Therefore, the demand of AI is increasingly transcending conventional diagnostic support applications to become a vital tool for uncovering the biological foundations of TCM formulas and syndromes [99].

#### 2.3.1 AI‐Driven Systems Pharmacology: From Network Mining to Mechanistic Hypothesis Generation

**FIGURE 3:**
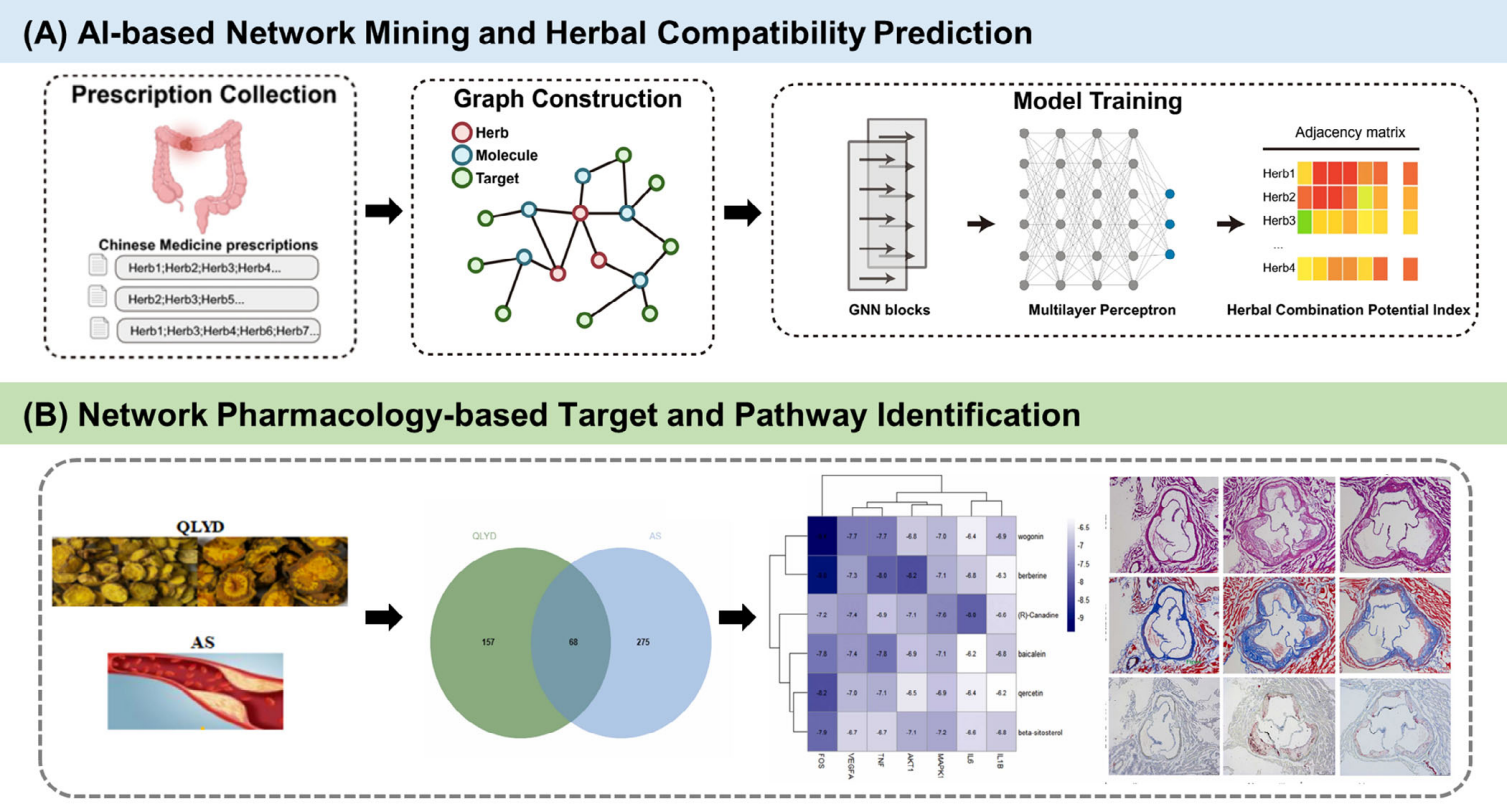
AI‐driven systems pharmacology for mechanistic hypothesis generation and validation in TCM. (A) Prediction of herbal compatibility for colorectal adenoma treatment based on graph neural networks. Copyright 2025, Springer Nature [103]. (B) Identification of bioactive compounds and potential mechanisms of scutellariae radix‐coptidis rhizome (QLYD) in the treatment of AS by integrating network pharmacology and experimental validation. Copyright 2023, Elsevier [104].

The introduction of AI has transformed traditional “ingredient–target‐pathway” analysis from static correlation to dynamic learning. Through ML algorithms such as GNN, systems pharmacology can automatically identify nonlinear relationships among key ingredients, action targets, and signaling pathways within complex TCM formula networks [100]. This enables the generation of experimentally verifiable mechanism hypotheses and the realization of precision TCM. For colorectal adenomas—high‐risk precancerous lesions—TCM formulas show promising effects, but understanding how the herbal combinations work and predicting their benefits is still challenging [101, 102]. Researchers gathered many treatment formulas and herbal ingredients. They built a neural network model to predict how strong herbal combinations are. Network pharmacology was also used to find key active ingredients. This approach helped explain how compound formulas work, offering a basis for improving TCM effectiveness, discovering new formulas, and supporting TCM for preventing and treating precancerous lesions (Figure 3A) [103]. In the treatment of atherosclerosis (AS) with Quyu Lixue Decoction (QLYD), researchers identified 49 active components and 225 corresponding targets of QLYD. A protein interaction network was constructed to screen for core genes (such as IL‐6, VEGFA, AKT1, TNF, and IL‐1β). GO and KEGG enrichment analyses revealed these targets primarily involve pathways related to inflammatory response, oxidative stress, and blood coagulation, including the TNF signaling pathway, IL‐17 signaling pathway, MAPK signaling pathway, and NF‐κB signaling pathway (Figure 3B) [104].

#### 2.3.2 Multiomics Integration for Decoding TCM Syndromes

The integration of multiomics technologies such as transcriptomics, proteomics, and metabolomics enables the construction of “gene–protein–metabolite” association networks. This approach reveals key biomarkers and signaling pathways associated with specific disease phenotypes, offering novel insights for elucidating disease mechanisms and identifying potential therapeutic targets. In recent years, the combined application of multiomics and computational biology has emerged as a significant research direction for elucidating the biological basis of TCM syndromes [105]. According to TCM theory, phlegm‐stasis (PBS) constitutes the pathological basis of CHD. Yang et al. systematically constructed a “gene–protein–metabolite” network for CHD with PBS syndrome (CHD‐PBS) by integrating transcriptomic, proteomic, and metabolomic data. Results revealed suppressed JNK/AP‐1 signaling and significant abnormalities in AA metabolism in CHD‐PBS patients, with core molecules including JNK1, FOS, CCL2, CXCL8, PTGS2, and CSF1. ELISA validation demonstrated that the combined CSF1 and JNK1 model exhibited the highest diagnostic efficacy (AUC > 0.93) [106]. In a study of ischemic heart failure (IHF) with Qi deficiency and blood stasis (QXXY) syndrome, researchers integrated transcriptomic, proteomic, and metabolomic data to construct a multiomics network for IHF with QXXY. This revealed that the syndrome is associated with energy metabolism disorders, chronic inflammation, and coagulation abnormalities. Key pathways include HIF‐1 signaling, glycolysis, and platelet activation. High‐diagnostic‐value biomarkers such as HIF‐1α, IL10, and SOD2 were identified, offering new insights into elucidating the molecular mechanisms underlying TCM syndromes [107].

#### 2.3.3 Data‐Driven Interpretation of Herbal Compatibility and Synergistic Mechanisms

AI‐driven systems pharmacology models are now expanding from the level of individual herbs to that of compound formulas. These models can predict interactions between primary constituents and core targets, and validate synergistic or antagonistic mechanisms within formulas through pathway enrichment analysis. This advancement is driving a shift from “empirical combination” to “mechanism‐guided combination” [108, 109]. Huang et al. constructed a driver network using multiomics data—including gene expression, proteomics, and methylation information—to identify core signaling pathway modules regulating key targets. Through this model, they predicted synergistic effects of different drug combinations and validated the mechanisms of these combinations via pathway analysis. This approach demonstrates a shift from “single‐drug target” to “combination intervention mechanism” analysis [110]. Niu et al. proposed a TCM Formula Prediction Model integrating TCM theory, AI algorithms, and network science. By combining herb scores based on target network importance, formula pairing scores derived from empirical learning, and formula prediction scores utilizing intelligent optimization and genetic algorithms, the model achieves efficient screening of optimal herbal combinations for diseases. The model successfully predicted optimal formulas with significant functional enrichment and network activity for Alzheimer's disease (AD), asthma, and AS, offering new strategies for mechanism elucidation and intelligent design of TCM formulas [111].

## 3 Nanotechnology‐Enabled Precision Delivery and Enhanced Efficacy of TCM Formulations

The four diagnostic methods, syndrome differentiation, and therapeutic treatment constitute the whole of the TCM diagnosis and treatment process. During the modernization of TCM, AI has significantly improved the accuracy and efficiency of TCM diagnosis by identifying, processing, and analyzing clinical data, providing a more scientific and reliable basis for further treatment. However, the modernization of TCM is not only to improve the accuracy of diagnosis, but also to improve the curative effect, including increasing drug absorption, improving drug distribution, reducing side effects, and so on. TCM mainly comes from natural medicines and their processed products, including plant medicines, animal medicines, mineral medicines, and some chemical and biological products. It has been discovered that the active ingredients extracted from TCM exhibited significant pharmacological activity and therapeutic potential for various diseases. Paclitaxel (PTX), artemisinin (ART), camptothecin, and triptolide (TP), which are both extracted from TCM, have been recognized as first‐line drugs for treating life‐threatening diseases or conditions that severely affect patients’ quality of life. However, most of the extracted ingredients are not suitable to be administrated directly due to their physicochemical properties, such as low permeability, instability, high hydrophilicity or hydrophobicity, which lead to undesired pharmacokinetic behaviors and limit the therapeutic efficacy in clinical [17]. Moreover, TCM formulas are characterized by “multicomponents, multitargets,” but their mechanism of action remains unclear. Figuring out the specific mechanisms and making the TCM formulas more controllable is an urgent issue that needs to be resolved in the process of modernization of TCM. Nanotechnology, as a cutting‐edge field in modern science, has shown immense potential in the modernization of TCM treatment [112].

**FIGURE 4:**
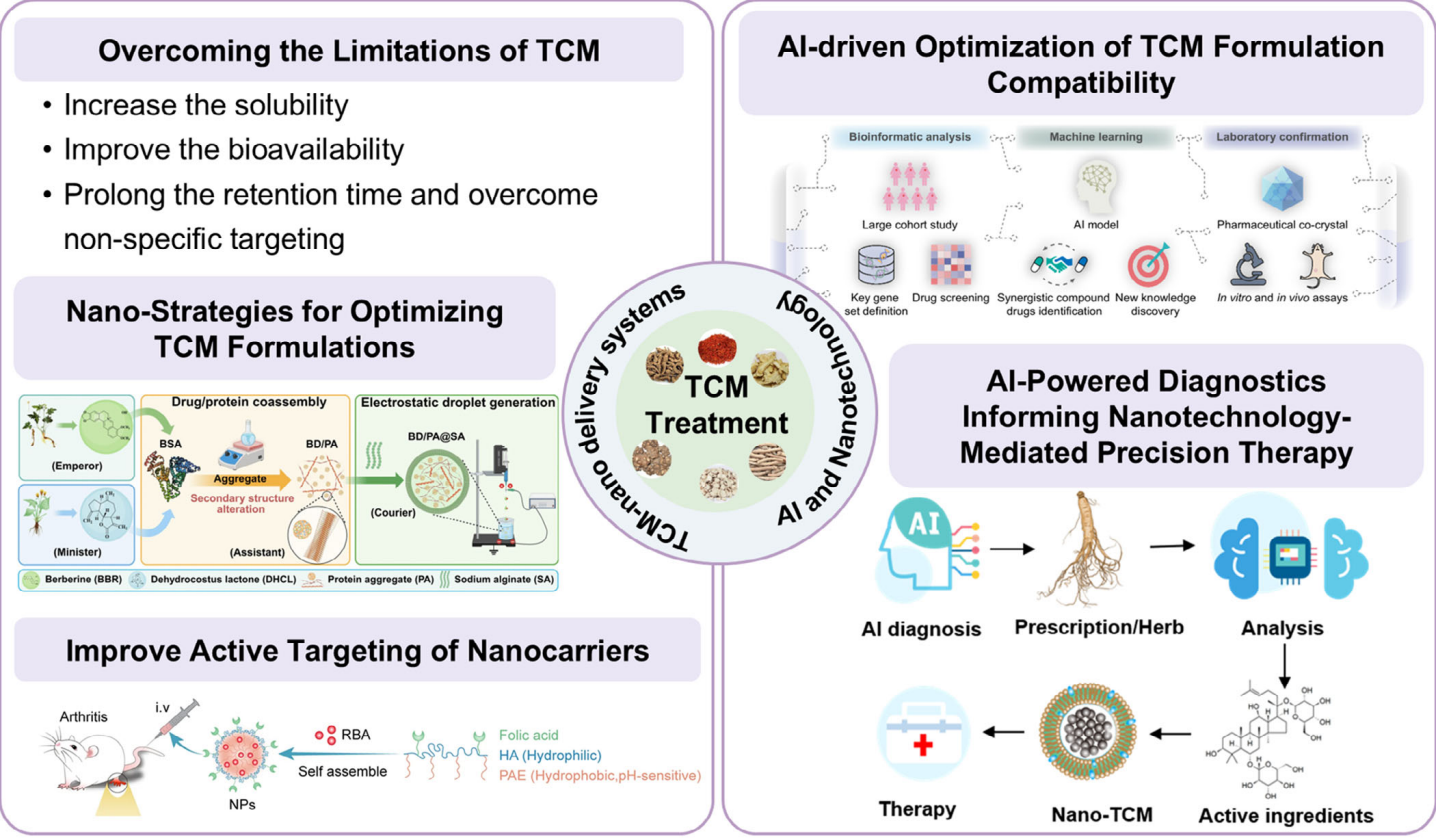
TCM treatment strategy based on nanotechnology and AI. Nanotechnology‐based delivery systems are employed to overcome intrinsic limitations of TCM. AI‐driven approaches integrate bioinformatic analysis, machine learning, and experimental validation to optimize formulation compatibility, identify synergistic components, and guide prescription selection. TCM treatments can be optimized through a closed‐loop system from AI‐powered diagnosis to nanotechnology‐assisted therapy.

Nano‐TCM refers to the use of nanotechnology to process the active ingredients (or active sites) of a particular component in a TCM formula into nanoparticles, or to load these active ingredients in nanocarriers (TCM‐nanodrug delivery systems) [113]. Nanotechnology not only accelerate the modernization of TCM but also overcome inherent limitations such as poor solubility, low bioavailability, and unclear pharmacokinetics of traditional preparations. Moreover, nanotechnology enables the rational design and optimization of TCM formulas, promoting synergistic therapeutic effects through controlled release and precise ratio modulation. In addition, by integrating functionalized nanocarriers or ligand modification strategies, nano‐TCM can enhance active targeting to specific tissues or pathological sites, thereby improving therapeutic efficacy while minimizing systemic toxicity (Figure 4).

### 3.1 Overcoming the Limitations of TCM

With the development of modern research in TCM, an increasing number of active ingredients have been discovered and confirmed to possess good pharmacological activity and efficacy, such as PTX, curcumin (Cur), and berberine (BBR). Due to the complex molecular structure, most TCMs or their active ingredients exhibit poor water solubility with high molecular weight, which significantly reduces their bioavailability and efficacy. In recent years, numerous studies have shown that nanotechnology can improve the solubility, permeability, and pharmacokinetic properties of TCM, thereby enhancing its bioavailability and facilitating clinical application.

#### 3.1.1 Increase the Solubility of the Active Ingredients of TCM

Most active ingredients of TCM exhibit poor water solubility, leading to low oral bioavailability, especially for class II drugs in the Biopharmaceutical Classification System. Since their bioavailability is closely related to solubility, nanotechnology can be employed to nanoformulate these compounds or encapsulate into nanocarriers, thereby improving their solubility and bioavailability.

**FIGURE 5:**
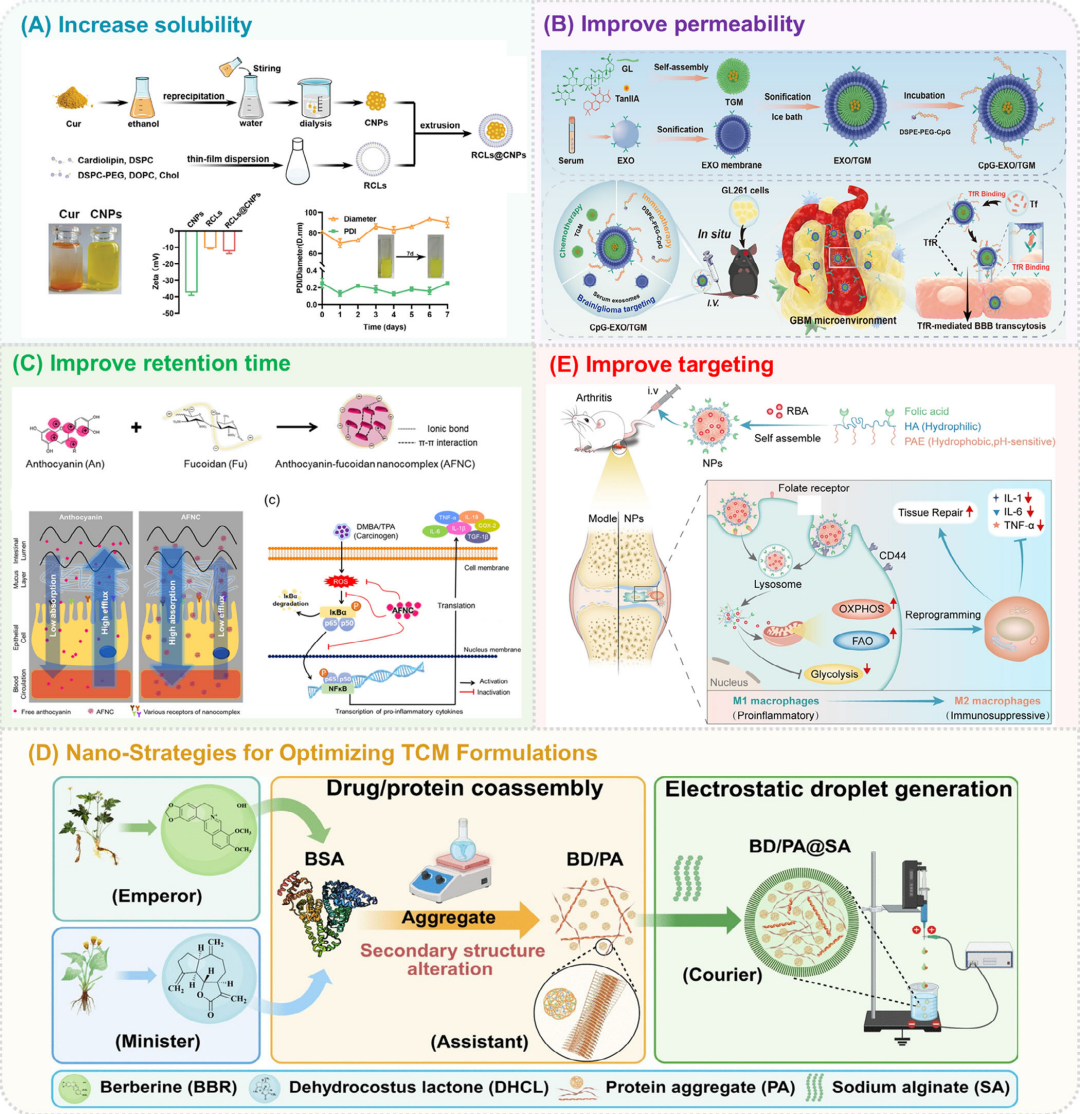
Application of nanotechnology to improve the efficacy and safety of TCM treatment. (A) Schematic diagram of responsive calloy phospholipid liposomes (RCLs)@ curcumin (Cur) nanoparticles (CNPs) (RCLs@CNPs) for improved solubility. Copyright 2024, Wiley. [118] (B) Schematic diagram of Toll‐like receptor 9 (CpG)–endogenous serum exosomes (EXO)/tanshinone IIA (TanIIA)–glycyrrhizic acid (GL) micelles (TGM) (CpG–EXO/TGM) for the blood–brain barrier (BBB) penetration. Copyright 2024, American Chemical Society [119]. (C) Schematic diagram of anthocyanin–alginate nanocomplexes (AFNC) for improved retention time in the gastrointestinal tract. Copyright 2020, Elsevier [120]. (D) Schematic diagram of roburic acid (RBA)‐nanoparticles (RBA‐NPs) for improved targeting ability of rheumatoid arthritis (RA). Copyright 2023, Springer Nature [121]. (E) Synthetic route of berberine (BBR) and dehydrocostus lactone (DHCL) (BD)/protein aggregates (PA)@sodium alginate (SA) (BD/PA@SA) designed according to the theory of “emperor”–“minister”–“assistant”–“courier.” Copyright 2025, Springer Nature [122].

The biological activity and pharmacological effects of TCM are related to their chemical structure and physical properties. Nanonization of TCM can be achieved through methods such as mechanical grinding, spray drying, and high‐pressure homogenization, which transforms the TCM to nanoscale to enhance solubility. Cur was reported to have antioxidant, anti‐inflammatory, antitumor, apoptosis‐inducing, and antiangiogenesis properties with extremely low toxicity [114]. However, the poor water solubility of Cur and its sensitivity to light and alkaline environments limit its effective concentration. Studies have shown that Cur's poor absorption and rapid metabolism lead to low bioavailability, with the bioavailability of orally administered Cur in rats being only 1% [115]. Kazunori Kadota et al. prepared Cur amorphous solid dispersion (ASD) composed of polyvinylpyrrolidone (PVP), α‐glucosyl stevia, and Cur by the freeze‐drying method with a sevenfold increased relative bioavailability, and the solubility of Cur in ASD was 2600‐fold higher than native Cur [116]. Furthermore, loading poorly soluble TCM active ingredients in nanocarriers is also an important strategy for increasing solubility. Chen et al. proposed a program of fabricating nanoscale γ‐cyclodextrin‐based metal–organic frameworks (nano‐CD‐MOFs) using a modified solvothermal method with the aid of surfactant PEG‐20,000 for the efficient delivery and protection of Cur [117]. Cur‐loaded nano‐CD‐MOFs dramatically increased solubility of Cur, nearly 2600‐fold higher than pure Cur and sixfold higher than Cur‐γ‐CD, and a top‐down uniform dispersion in the dissolution process. Similarly, self‐assembly of Cur molecules into carrier‐free pure Cur nanoparticles (CNPs) can also improve the application limitations of Cur. Feng et al. used a simple and green reprecipitation method to synthesize CNPs [118]. CNPs are loaded into reactive oxygen species (ROS) responsive cardiolipin liposomes (RCLs) to obtain RCLs@CNPs (Figure 5A). The hydrophobic Cur molecules self‐reassembled into CNPs via intermolecular interactions (e.g., hydrophobic interactions) during solvent‐exchange process. Compared with water‐insoluble Cur, CNPs dispersed well in aqueous solution thanks to the large surface area of nanoparticles, which was expected to improve bioavailability and efficacy.

#### 3.1.2 Improve the Bioavailability of the Active Ingredients of TCM

Many active ingredients of TCM are limited in their therapeutic efficacy due to poor bioavailability, which can arise from low membrane permeability, instability, or physiological barriers. Nanotechnology exhibits the potential to enhance their bioavailability. BBR is widely used in food and medicine due to its low toxicity and multiple activities, including anti‐inflammatory, antibacterial, antidiabetic, and antitumor properties. However, as an isoquinoline alkaloid, BBR exhibits poor pharmacokinetic properties and strong hydrophobicity due to its chemical structure, which limit its clinical application with stability and intestinal permeability [123]. The cationic groups in BBR's structure have a high affinity for the multidrug efflux pump P‐glycoprotein (P‐gp) in the gastrointestinal tract [124]. Nanodelivery systems, such as lipid nanoparticles,[125, 126, 127, 128] polymers,[129, 130, 131, 132, 133, 134] and metal nanoparticles [135, 136, 137] have been exploited to enhance BBR's bioavailability. Li et al. designed a BBR hydrochloride nanoemulsion system and further evaluated its intestinal permeability using Caco‐2 cells. The results showed that the permeability of the BBR hydrochloride nanoemulsion was significantly increased, and the efflux by P‐gp was significantly reduced, demonstrating good oral bioavailability [124].

Transdermal and transmucosal administration are common methods for delivering TCM. Active ingredients are absorbed through the skin and mucosa into the systemic circulation to exert systemic or local therapeutic effects. However, the permeability of these ingredients is often hampered by the barrier functions of the skin and mucosa. Here again, limited permeability translates to reduced bioavailability. Aconitine (ACO) is often used to treat pain, RA, and other inflammatory conditions. However, it has high cardiovascular toxicity that can lead to life‐threatening arrhythmias and cardiac arrest, which limits its broader medical application. Guo et al. developed dissolving microneedles (MNs) loaded with ACO in solid lipid nanoparticles to enhance transdermal delivery by disrupting the barrier function of the stratum corneum and allowing sustained drug release, significantly increasing drug permeability in the skin while reducing toxicity [138]. Additionally, the area under the concentration–time curve of ACO loaded by MNs was 1.95‐folds higher than that of solid lipid nanoparticles applied to the skin.

Nanotechnology has also shown great potential in overcoming the blood–brain barrier (BBB). BBB maintains and regulates central nervous system (CNS) homeostasis by tightly controlling the movement of substances between the blood and the brain, including the exchange and transport of molecules, ions, or cells. Notably, more than 98% of small molecule drugs and almost 100% of large molecule drugs fail to reach the brain through the circulatory system. Nano‐TCM can provide a more effective therapeutic platform for treating CNS diseases, targeting drug delivery to cells and tissues within the CNS, and achieving slow and controlled release of drugs in the brain. Cui et al. constructed tanshinone IIA (TanIIA)–glycyrrhizic acid (GL) micelles (TGM) by the self‐assembly strategy of TanIIA and GL, and loaded them in serum EXO (Figure 5B) [119]. Endogenous serum exosomes are selected to coat the pure drug nanomicelles, and the CpG oligonucleotides, agonists of Toll‐like receptor 9, are anchored on the exosome membrane to obtain immune exosomes loaded with TCM self‐assembled nanomicelles (CpG‐EXO/TGM). The CpG ODN was coupled with phospholipid and anchored on the EXO membrane to construct CpG‐EXO/TGM that could realize the codelivery of TGM and CpG ODN. After intravenous injection of CpG‐EXO/TGM, TfR could bind to free Tf in blood, and then CpG‐EXO/TGM traversed the BBB through TfR‐mediated transcytosis.

#### 3.1.3 Prolong the Retention Time and Overcome Nonspecific Targeting of the Active Ingredients of TCM

Nanotechnology‐mediated prolongation of retention time is not only crucial for prolong the retention time but also an important means of overcoming the challenge of nonspecific targeting by controlling the physicochemical properties of nanoparticles. With increased retention time and stimulus‐responsive properties, nanocarriers enable active ingredients to preferentially accumulate at pathological sites, thereby enhancing therapeutic efficacy while minimizing off‐target effects.

Many active ingredients in TCM suffer from insufficient accumulation in the body and short retention times, which lead to inadequate drug concentrations and significantly affect their efficacy. Anthocyanins and their glycosides are water‐soluble, nontoxic natural compounds with significant antioxidant, anti‐inflammatory, antimutagenic, antidiabetic, and anticancer properties. However, due to their high sensitivity to environmental factors (ascorbic acid, light, metal ions, oxygen, and pH), processing (heat), and gastrointestinal digestion processes (enzymes, proteins, and pH), anthocyanins, and their glycosides exhibit poor stability and are easily degraded. Li et al. chose alginate (an anionic polymer) to produce anthocyanin–alginate nanocomplexes (AFNC), which enhanced gastrointestinal absorption and chemical stability through ionic bonds and π–π stacking between anthocyanins (Figure 5C) [120]. This approach helps prevent the rapid metabolism and excretion of free anthocyanins in the body, thereby prolonging their retention time in the gastrointestinal tract.

Moreover, the retention and accumulation of active ingredients at target sites can be further improved by precisely regulating the size of nanoparticle. According to drug combination of TCM, *Coptidis rhizome* and *Lonicera japonica* are a pair of herbs to clear away heat and toxins. BBR, a kind of alkaloid from *Coptidis rhizome* and chlorogenic acid, a type of phenolic acid from *Lonicera japonica* are chosen to self‐assemble into herbal‐derived nanoparticles through noncovalent electrostatic, π–π, and hydrophobic interactions [139]. These nanoparticles could maintain stable structures under normal physiological conditions (pH 7.4) as well as in the presence of fetal bovine serum. Under acidic conditions that mimic the microenvironment of osteoporosis (pH 4.0), nanoparticles transformed into larger and unstable particles, which facilitated their retention and drug release within the bone tissue.

### 3.2 Nanostrategies for Optimizing TCM Formulations

TCM formulas are characterized by their inherent complexity, often consisting of multiple bioactive components that exert synergistic therapeutic effects through multitarget interactions. Nanotechnology offers an attractive solution by enabling the coencapsulation and controlled delivery of multiple herbal ingredients in a single carrier system. Advanced nanostrategies such as liposomes, polymeric nanoparticles, micelles, and hybrid nanoplatforms not only improve the pharmacokinetic behavior of individual compounds, but also facilitate the simultaneous delivery of multiple active ingredients, thereby preserving the holistic therapeutic concept of TCM while enhancing its precision and efficacy. Zhao et al. developed a multifunctional hydrogel system composed of three natural polymers: oxidized astragalus polysaccharide (OAPS)–carboxymethyl chitosan (CMC)–sodium methacrylated alginate (SAMA) cross‐linked with magnesium ions (Mg^2+^) (OAPS–CMC/SAMA–Mg^2+^, abbreviated as OCS) [140]. This hydrogel was further integrated with *Achyranthes bidentata*‐derived supramolecular self‐assembly (NX SSA) loaded with Cur (NX@Cur), forming a composite designated as OCS/NX@Cur. OCS/NX@Cur achieved a synergistic therapeutic effect differentiated from that of conventional synthetic carriers through a graded controlled release. The noncovalent interaction between the hydrogel network and NX SSA not only inhibited the sudden release of the drug but also rapidly neutralized the ROS in the inflammatory microenvironment (13 vs. 70% at 6 h) [141, 142]. Under the trigger of the acidic microenvironment of the wound, the sustained slow hydrolysis of NX SSA prolonged the release cycle to five times that of the conventional carriers (240 vs. 48 h), allowing the drug release kinetics to be precisely matched with the inflammatory process.

Based on the TCM theory of “Jun” (emperor), “Chen” (minister), “Zuo” (assistant), and “Shi” (courier), Jiang and colleagues selected BBR and dehydrocostus lactone (DHCL) (abbreviated BD) as compatible therapeutic drugs, where BBR played an “emperor” role and DHCL played a “minister” role (Figure 5D) [121]. Subsequently, protein aggregates (PA, composed of nano/microscale oligomers and fibrils), which played an “assistant” role, loaded BD to form BD‐embedded protein aggregates (BD/PA) through the drug/protein coassembly technique. Finally, BD/PA encapsulated by sodium alginate (SA) microspheres (BD/PA@SA) was prepared via electrostatic droplet generation, with SA microspheres (@SA) serving a “courier” role. Therefore, natural product‐derived BD/PA@SA was formed via the above hierarchical assembly process for targeted UC therapy.  The entire formulation exhibited anti‐UC efficacy through a three‐pronged strategy, including mitigating inflammation, repairing the mucosal barrier, and modulating the gut microbiota.

These TCM‐nano highlight how the codelivery of multiple herbal ingredients within polymeric or hybrid carriers can not only improve pharmacokinetics and therapeutic precision, but also embody the holistic principle of TCM. Such approaches bridge traditional theory with modern nanotechnology, offering promising avenues for the development of next‐generation TCM formulations with enhanced efficacy and controllability.

### 3.3 Active Targeting of Nanocarriers for Enhanced Therapeutic Efficacy and Reduced Off‐Target Effects

Nano‐TCM can utilize targeting ligands such as antibodies or peptides to specifically direct active ingredients to particular cells and tissues, which can enhance their specificity for pathological tissues or cells and reduce systemic toxicity. This targeted approach enables TCM to accumulate at high concentrations in the action site, thereby improving therapeutic efficacy.

TP possesses various biological activities, including anti‐inflammatory, antitumor, and immunomodulatory effects. Zheng et al. designed and synthesized hyaluronic acid (HA)‐modified cationic liposomes (TPLP‐HA) to load TP for targeted breast cancer therapy [143]. The results showed that TPLP‐HA exhibited significant in vivo antitumor effects on breast cancer and TPLP‐HA significantly reduced nephrotoxicity and hepatotoxicity compared with free TP. Roburic acid (RBA), an ingredient from anti‐RA herb *Gentiana macrophylla* Pall., displayed strong anti‐inflammatory activity. However, its medical application is limited by its hydrophobicity, lack of targeting capability and unclear functional mechanism. Jia et al. constructed a pH responsive dual‐target drug delivery system hitchhiking RBA (RBA‐NPs) that targeted both CD44 and folate receptors (Figure 5E) [122]. In rat RA model, the nanocarriers effectively delivered RBA to inflammatory sites and significantly enhanced the therapeutic outcomes compared with free RBA, as well as strongly reducing inflammatory cytokine levels and promoting tissue repair.

Moreover, TCM and its active ingredients can be delivered controllably to the target site under external stimuli if the delivery carriers are designed with special characteristics. Resveratrol (Res) is a natural polyphenol with various pharmacological activities, including cardio protection, platelet disaggregation, antioxidant, anti‐inflammatory, and vasodilatory properties. Abbas et al. aimed to improve the efficacy of Res for treating AD via the nasal olfactory mucosa for brain targeting [144]. They prepared chitosan coated bilosomes (nonionic surfactant vesicles stabilized by bile salts), loaded with Res and superparamagnetic iron oxide nanoparticles (SPIONs), and incorporated into SA/PVP wafers. SPIONs loaded bilosomes could be guided more precisely to the olfactory nerve endings by applying an external magnetic field. Results revealed improved memory and cognitive functions in LPS induced AD mice, with the down regulation of NF‐κB, P38, and proinflammatory markers.

## 4 The Convergence of AI and Nanotechnology: Paving the Way for Personalized TCM

TCM formulas are multicomponent systems built on the principles of syndrome differentiation and treatment. The core of these formulas lies in the synergistic therapeutic effects of various components on the human body, which is originated from the TCM theory of “Jun” (emperor), “Chen” (minister), “Zuo” (assistant), and “Shi” (courier). Understanding the combination rules of TCM formulas through modern scientific methods can help reveal their underlying mechanisms and provide new or more effective formula guidance for practitioners in clinical practice. With the rapid development of AI, nanotechnology and other technologies, the mechanism of action of TCM formulas is being revealed and explained scientifically. Moreover, the AI‐powered diagnosis can provide information of patients’ constitution and biomarkers, forming a closed‐loop system for precision therapy.

### 4.1 AI‐Guided Design and Optimization of Nanodelivery Systems for TCM

Most TCM formulas are in the form of decoctions, where the phytochemicals from herbs or plant materials dissolve in boiling water to form a complex system with multiple phases, including molecules, nanoaggregates, precipitates, and emulsions. In recent years, several studies reported that it was the nanoaggregates in decoctions that play a key role in mediating the absorption, transport, and bioavailability of active phytochemicals. For instance, Zhang et al. identified 21 major components in T‐QY305 and 13 in its nanoparticle fraction (N‐QY305) by LC–MS, confirming that nanoaggregates concentrate bioactive compounds with characteristic UV absorption patterns [145].

**FIGURE 6:**
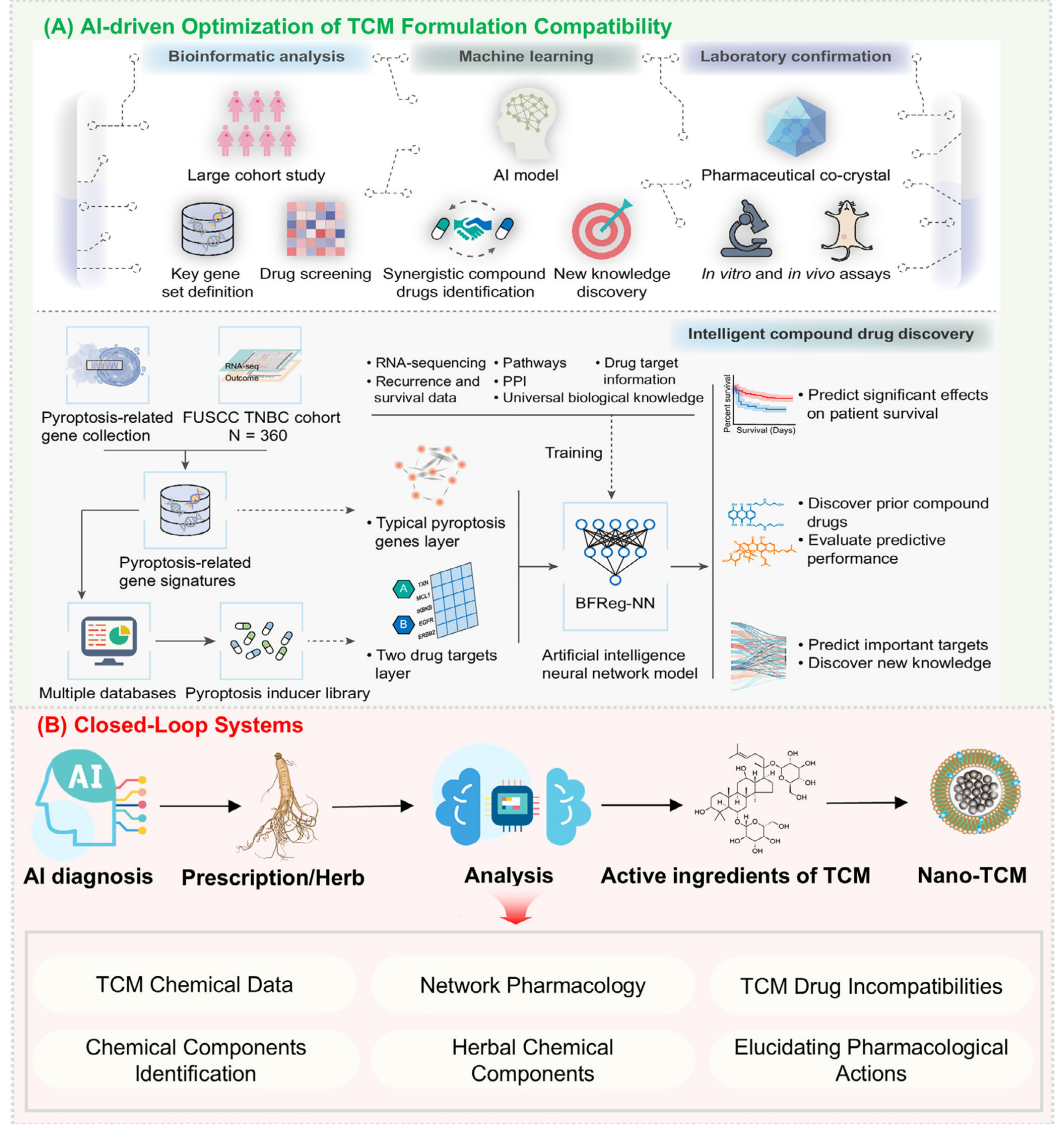
The convergence of AI and nanotechnology: paving the way for personalized TCM. (A) The workflow for discovering and optimizing antitriple‐negative breast cancer (TNBC) compound pyroptosis drugs based on big data and artificial intelligence. Copyright 2023, Springer Nature [151]. (B) AI‐driven diagnosis, real‐time data collection, and intelligent nanocarriers form a dynamic feedback loop, enabling personalized and adaptive treatment plans.

In recent years, the paradigm of natural product research and development has been transformed by advances in AI. The integration of multiple ML models and neural network architectures has substantially shortened the drug discovery cycle [146, 147, 148, 149]. The AI‐guided design and optimization of TCM‐based nanodelivery systems hold great promise for accelerating drug discovery, improving formulation performance, and driving the modernization of TCM. Zhang et al. developed a BERT‐based molecular antioxidant property prediction model and applied it to identify potential candidate compounds from natural herbs with about 20% higher accuracy compared with conventional ML models (RF and SVM) [150]. This method uses a transformer architecture to successfully capture the underlying features of the SMILES structure of antioxidant compounds and autonomously learns to screen for novel compounds with potential antioxidant properties in a natural herbal compound library. Finally, based on the attention weight analysis, the compounds receiving the highest scores were identified as the key active constituents corresponding to the main herb in TCM theory. Three representative compounds (5,7‐diacetoxy‐8‐methoxyflavone, fisetin, and honokiol) were further encapsulated into functional liposomes to validate their antioxidant performance in vitro and in vivo. The results indicate that the liposomal delivery system not only enhanced in vivo bioavailability, but also mitigated oxidative stress injury after kidney acute ischemia/reperfusion. Ouyang et al. further illustrated the potential of AI in guiding rational drug combination and nanomedicine development [151]. They used a case study of necroptosis therapy for triple‐negative breast cancer (TNBC) to leverage data mining from a large TNBC cohort and a drug database (Figure 6A). They performed a bioinformatic analysis of the typical pyroptosis genes to generate target omics associated with TNBC and then identify corresponding potential pyroptosis‐inducing drugs based on a large TNBC cohort and drug databases. Subsequently, a biofactor‐regulated neural network AI model, BFReg‐NN, is established to screen and optimize pyroptosis compound drugs rapidly. Ultimately, they prepared a biomimetic nanocrystal of a selected combination of mitoxantrone and garcinia acid for efficient drug delivery. In the TNBC model, the obtained nanocrystal revealed a unique mechanism that regulated necroptosis genes and triggered necroptosis‐associated immune effects through ribosomal stress. This work explored an innovative drug development paradigm based on a target–omics framework for intelligent compound discovery, repurposing existing drugs to precisely treat refractory diseases. The smart data analysis and drug screening not only improved the selection efficiency of drug combinations but also provided new insights for the development of modern TCM. Compared with traditional empirical methods, AI‐driven data approaches can uncover the synergistic mechanisms of TCM formulas more rapidly and accurately. Additionally, the comprehensive use of big data and ML algorithms allows for a more systematic analysis of the pharmacological mechanisms of TCM formulas, optimizing their compatibility and improving clinical efficacy.

### 4.2 Closed‐Loop Systems: AI‐Powered Diagnostics Informing Nanotechnology‐Mediated Precision Therapy

The integration of AI and nanotechnology is drawing a promising future of precision therapy from diagnosis to treatment. The dynamic feedback loop formed by AI‐driven diagnosis, real‐time data collection, and intelligent nanocarriers enables personalized and adaptive therapy (Figure 6B).

Starting from disease analysis, AI algorithms can analyze large‐scale multiomics data, clinical imaging, and even specific diagnostic information in TCM (such as syndrome differentiation), to identify key molecular targets and patient‐specific pathological patterns [152, 153, 154, 155, 156]. In the context of TCM, AI helps identify disease biomarkers, predict patient‐specific responses, and understand the multifaceted interactions in TCM formulas. This approach aligns with the holistic principles of TCM and provides a scientific basis for its treatment strategies. Lin et al. analyzed the data of 261 colorectal cancer (CRC) cases recruited from a total of 141,962 cases of renowned veteran TCM doctors collected from datasets of both the DeepMedic software and TCM cancer treatment books. Over 20% of CRC patients demonstrated symptoms of poor appetite, fatigue, loose stool, and abdominal pain. By analyzing the formula patterns of TCM, they found that Atractylodes macrocephala (Bai‐zhu) and Poria (Fu‐ling) were the most commonly prescribed single herbs identified through analysis of medical records and supported by the neural network analysis [157].

In addition, nanosensors integrate different nanomaterials with sensing elements to form biosensing devices that can detect biological or chemical targets [158]. Then, AI can analyze this information and identify specific biomarkers, thereby helping to determine the most effective treatment plan [159, 160, 161, 162]. Combining the advantages of nanosensors with AI technology has resulted in AI‐assisted nanosensors, providing intelligent, rapid, and highly sensitive tools for clinical diagnosis and treatment. The SimpleSense‐BP of NanoWear is currently the first and only wearable nanosensor approved by the US FDA for AI assistance [163]. This device provides real‐time, clinical‐level cardiovascular data for continuous blood pressure monitoring. This data‐driven approach enables clinicians to design personalized treatment plans, increasing the likelihood of achieving better health outcomes.

However, there are currently no studies that can directly apply AI diagnosis or public case data analysis to guide the design of nanocarriers. Network pharmacology can be utilized to construct the interactions between herbs, their active components and biological targets, thereby gaining a deeper understanding of the mechanism of action of TCM formulas and facilitating the rational design of treatment strategies. Zhou et al.’s recent research indicated that AI‐based analysis can effectively predict the compound‐target networks in TCM preparations, providing a theoretical basis for converting complex herbal formulas into quantifiable nanodelivery strategies. While the nanodelivery lacks the guidance of accurate diagnosis driven by AI for personized treatment [164].

### 4.3 Future Vision of Integrated AI‐Nano Platforms for TCM

The integration of AI and nanotechnology will completely transform the modernization and personalization processes of TCM. The future AI‐nanotechnology platform will go beyond the traditional delivery and disease monitoring models of TCM, establishing an intelligent and adaptive ecosystem.

The main direction of this transformation is AI‐driven drug discovery and formula design, including advanced computational methods such as DL, network pharmacology, and systems biology, which will be able to interpret the multicomponent and multitarget interactions in complex TCM formulas [165, 166, 167]. By combining these predictive models with molecular docking and TCM chemical databases, researchers will be able to design nanocarriers for specific herbal combinations, thereby enhancing the synergistic effect while reducing toxicity [168, 169]. Therefore, the AI‐guided formula process can be screened, optimized, and verified, shortening the research and development cycle of nano‐TCM and improving the conversion efficiency.

Another emerging frontier area is the self‐adaptive and responsive nanoplatforms, which integrate biological sensing and AI‐based feedback mechanisms. With the recognition capabilities of these systems, the treatment procedures can be promptly adjusted to adapt to the patient's responses and the changes in the disease progression. Wang et al. applied a powerful phenotypically driven platform, termed feedback system control, that systematically and rapidly converges upon a combination consisting of three nanodiamond‐modified drugs (nanodiamond–doxorubicin, nanodiamond–mitoxantrone, nanodiamond–bleomycin) and unmodified PTX that is simultaneously optimized for efficacy against multiple breast cancer cell lines and safety against multiple control cell lines [170]. The results showed that AI‐optimized nanomedicine drug combinations performed better than randomly selected combinations, AI‐optimized unmodified drug combinations, and monotherapies. This preemptive strategy guarantees that patients get the most effective and personalized care throughout their treatment journey.

At the same time, the integration of multimodal data and digital twin technology will redefine the scope of TCM research and practice. It can integrate genomic, metabolomic, digital imaging, wearable sensor data, and AI algorithms for clinical indicators to construct personalized digital twins [171, 172, 173, 174]. These models can simulate the development process of diseases, predict the body's response to TCM nanoparticles, and provide real‐time guidance for treatment adjustment plans. Within this framework, the traditional concepts of “diagnosis and treatment based on syndrome differentiation” and “holistic regulation” in TCM can be mapped onto personalized digital health records, thus achieving the integration of TCM theory with modern computational medicine.

However, there are still many challenges in achieving this goal. The data standardization work between TCM formulas has not been completed, and the interpretability and ethical governance of AI algorithms also require special attention. As research gradually adopts a comprehensive treatment model, AI and nanotechnology platforms are leading the transformation of TCM into a scientifically rigorous and personalized platform.

## 5 Challenges, Clinical Translation, and Future Perspectives

### 5.1 Technical and Regulatory Hurdles: Data Quality, Model Interpretability, Nanotoxicology, and Standardization

Although significant advances have been made in applying AI and multiomics to TCM, several technical and regulatory barriers hinder clinical translation. Insufficient data quality and lack of standardization remain the primary limitations for model generalization. The four diagnostic methods in TCM involve the collection of multimodal information, including tongue appearance, pulse characteristics, complexion, and voice. This information exhibits high complexity and individual variability, while the collection process is influenced by factors such as environmental conditions, instrumentation, and operational methods. Currently, there is no unified standard for the collection process of the four diagnostic methods. Aspects such as the angle for photographing tongue coating, light intensity, pulse measurement pressure, and frequency lack standardized requirements [175]. For example, different researchers use varying equipment and classification methods for tongue diagnosis images, leading to significant differences in data quality between laboratories or clinical institutions [176, 177]. Although researchers have established the TCMEval‐SDT benchmark dataset to evaluate algorithm and model capabilities in TCM syndrome diagnosis, this dataset remains small in scale, containing only four case histories related to Qi, blood, and body fluid disorders [178]. The application of instrument‐assisted TCM diagnostic systems in clinical settings also faces limitations. For example, sensors used in pulse diagnosis may struggle to record subtle changes and exclude the influence of the subject's respiratory movements [179]. Furthermore, AI models constructed in some studies lack interpretability, making it difficult to clearly establish the correspondence between model judgments and the characteristics of the four diagnostic methods. For example, Chen et al. proposed a DL‐based automated tongue pattern analysis system. Although this system demonstrated good performance and robustness in feature extraction and classification, it lacked transparency and understandability regarding the model's decision‐making process [180].

Future research should advance the standardization of data collection processes for four diagnostic methods in TCM and establish large‐scale, multicenter, publicly shared databases to mitigate the impact of individual and inter‐laboratory variations on model performance. Concurrently, integrating multimodal information such as tongue patterns, pulse patterns, complexion, and voice can construct unified feature representations. Combining these with AI algorithms to optimize diagnostic models, while prioritizing model interpretability, ensures clear correspondence between AI decisions and Four Diagnostic Methods features. This approach enhances clinicians' understanding and trust in algorithm outputs. Ultimately, developing intelligent auxiliary systems capable of real‐time data collection, analysis, and feedback—coupled with clinical validation—can provide scientific grounds for modernizing and clinically promoting the four diagnostic methods of TCM.

Nanotechnology has to some extent reduce the toxic effects of TCM. However, the toxicity of nanomaterials still hinders the translation to clinical practice. Some synthetic materials such as cationic polymers and metal nanoparticles may cause cellular stress, inflammatory responses or immune activation in the body, and even affect long‐term tissue functions [181, 182]. Liposomes have become the main category of nanomedicines approved by the US FDA due to their multifunctionality and effectiveness [183]. They are typically composed of phospholipids and can form single or multilayer vesicle structures, enabling them to effectively carry and deliver a variety of drugs, including both hydrophilic and hydrophobic compounds. Zhang et al. developed a Celastrol (CEL, a key active ingredient obtained from *Tripterygium wilfordii*)‐loaded ginsenoside Rg3 (Rg3) liposome (CR‐Lip) designed to precisely target tumor cells in obesity‐related ITME while maintaining robust immunological activity [184]. The study demonstrated that CR‐Lip, incorporating CEL, did not exhibit significant adverse effects at a dose of 2 mg/kg. Toxicity assessments revealed no notable variations in liver, kidney, or cardiac functions, and histological evaluations showed no discernible damage to major organs. In addition, the new drug delivery system relies more on polymer carrier materials, which plays a crucial role in promoting the innovation of drug formulations and the development of drugs. Polymer carriers have various drug loading modes, including covalent coupling to form polymer–drug conjugates, polymer micelle encapsulation of drugs, polymer vesicle encapsulation of drugs, and drugs dispersed in polymer gels [14]. Wang et al.’s research aims to covalently conjugate the borneol molecule with US FDA‐approved polyethylene glycol (PEG) as hydrophilic blocks (methacrylate–PEG–borneol) and with ROS‐cleavable thioketal (TK) bond linkers as hydrophobic blocks (methacrylate–TK–borneol) [185]. Multiple findings indicate that the formulated preparations have high biocompatibility. Furthermore, self‐assembling nanosystems are mostly constructed from the drugs themselves or natural polymers, avoiding the use of exogenous additives, which results in better overall biocompatibility and further reduces the risk of toxicity [186, 187, 188]. Zhang et al. focused on a TCM formula T‐QY305, also known as Qi Yin San Liang San Decoction, that can modulate the recruitment of neutrophil in skin and colon tissue, and was found to be effective in alleviating cutaneous adverse reaction and diarrhea caused by epidermal growth factor receptor inhibitors [145]. The nanostructure N‐QY305, further isolated from T‐QY305, exhibited higher potency in reducing adverse reactions than the original decoction. During the self‐assembly process of preparing TCM nanoparticles, no additional nanocarriers are involved. This not only enhances the drug loading efficiency (even up to 100%), but also avoids the toxicity of the nanocarriers and promotes large‐scale scalable production and clinical translation [186, 187, 188].

### 5.2 Bridging the Gap: Requirements for Robust Clinical Validation

Although DL and multimodal fusion technologies have significantly improved the performance of TCM diagnostic systems, their application in clinical practice is still in its early stages. Currently, most models are trained and validated using small sample, single‐center datasets, lacking prospective cross‐center studies to verify their reproducibility in different populations and clinical environments. Moreover, many studies rely on retrospective data without clinical annotations, which limits the credibility of the models in actual clinical diagnoses [189]. Therefore, establishing a unified evaluation index system, such as diagnostic sensitivity, specificity, and consistency with expert diagnoses, is of vital importance for evaluating the clinical value of the model. At the same time, the AI model must be interpretable, so that the clinical features it outputs can match those obtained from the four diagnostic methods of TCM, and can explain the algorithmic decisions to clinical doctors [190]. Therefore, the key to promoting clinical transformation lies in truly embedding the AI diagnostic system into the clinical workflow. This requires achieving interoperability with hospital information systems and electronic medical record systems, and establishing standardized data exchange protocols to support real‐time decision‐making. The collaboration among AI, clinicians, and regulatory agencies is crucial for developing validation frameworks that comply with international standards (such as ISO/IEC 62304 and the “good machine learning practice” of the US FDA) [191]. Ultimately, only through large‐scale, prospective, and clinically supervised research can the AI‐based four diagnostic methods of TCM evolve from experimental prototypes to reliable and precise clinical tools in TCM.

Based on clinical validations of various active ingredients such as ART, PTX, and BBR, the potential use of TCM as a source of clinical drugs is increasingly being accepted. Although many nanodelivery formulations have received US FDA approval or completed Phase III clinical trials (mainly for cancer and neurological diseases) [192], only one TCM active ingredient (PTX), has been successfully marketed as nanoformulation. Lipusu (PTX liposome) was first reported to be approved for market in China in 2003 for the treatment of various solid tumors. In addition, there are other four PTX nanomedicines has been approved: Abraxane, Genexol PM, Nanoxel M, and Paclical (Apealea). The US FDA approved Abraxane for the treatment of metastatic breast cancer in 2005. Genexol PM was the earliest polymer nanoparticle drug approved for human use in South Korea, the Philippines, India, and Vietnam in 2007, with indications including metastatic breast cancer, non‐small cell lung cancer, and OC. Nanoxel M is a PTX polymer microsphere used for cancer treatment, which was approved in 2007 in India. Paclical was approved in Russia for the treatment of OC in 2015. NK105 (Nippon Kayaku et al., polymer‐micelle nanoparticle PTX) has conducted multinational Phase I–III trials (including a multinational III comparison trial for metastatic/relapsed breast cancer) (NCT01644890). Most of the reported nanomedicine systems have shown promising efficacy in animal models, but they have not yet entered human trials [193]. The difficulties in this transformation stem from the lack of TCM pharmacokinetics, unclear mechanisms, and the absence of standardized preclinical evaluation standards [194]. Therefore, establishing a repeatable and strict clinical validation framework is crucial for obtaining regulatory approval and final clinical implementation. For multicomponent TCM formulas, the multitarget and synergistic mechanisms of their components make the identification of active ingredients and the dose‐response relationship complex. When these complex mixtures are incorporated into nanocarriers, the interactions between herbal components further affect the stability, release kinetics, and biological performance of the nanoparticles. Thus, the traditional pharmacokinetic and toxicological assessment frameworks for single‐compound drugs are insufficient to predict the overall behavior of nanomedicine formulations. Moreover, converting nanotechnology into commercial products is a challenging and costly task. It takes approximately 8 years and 7.5 billion US dollars to develop and commercialize a new type of anticancer nanomedicine and only less than 50% of the candidate drugs that enter the phase III clinical trial eventually receive approval from the US FDA [195, 196]. Due to the increased complexity of herbal sources, ambiguous regulatory regulations, and the lack of internationally recognized clinical standards, the obstacles faced by nano‐TCM are even higher.

To bridge this gap, a comprehensive framework that integrates AI‐assisted pharmacokinetic modeling, multiomics data, and network pharmacology will play a crucial role in optimizing the selection of candidate drugs and predicting the feasibility of transformation. Ultimately, the success of the clinical validation of nanomedicines will depend on interdisciplinary collaboration, standardized data, and regulatory guidelines for the uniqueness of nanomedicines derived from TCM.

### 5.3 Future Research Directions: Intelligent Responsive Nanosystems and Large‐Scale Real‐World Evidence

Future research in nano‐TCM is expected to develop toward the intelligent, adaptive and stimulus‐responsive nanosystems that can respond to the dynamic regulation of drug release in the pathological microenvironment. By integrating microenvironment response mechanisms such as pH sensitivity, redox gradients, enzyme activity, or ROS levels, these intelligent nanosystems can achieve the controlled delivery of active ingredients from TCM [197, 198]. Nanomedicine will also further develop into an autonomous therapeutic platform that can self‐optimize dosage and release kinetics through real‐time monitoring of biological signals or remote control based on AI algorithm. The fusion of AI and nanotechnology will not only enhance accuracy but also align with the holistic concept of individualized treatment in TCM.

Real‐world evidence (RWE) originates from data collected in routine clinical practice rather than randomized controlled trials, and it is becoming increasingly important in evaluating the safety, efficacy, and usage patterns of TCM. The complexity, individualization, and multicomponent nature of TCM methods pose unique challenges to traditional randomized controlled trial designs, while real‐world studies (RWS) offer the opportunity to capture large‐scale real clinical practice and patient diversity. In recent years, the National Medical Products Administration under the “Classic Formula Restoration” policy has approved hundreds of classic TCM decoctions and formula granules for clinical use [199]. A national multicenter post‐marketing study involving 30,400 patients evaluated the cardiovascular indications of Danshen Dihuang Injection (based on Salvia miltiorrhiza), showing good safety and usage patterns in different clinical settings [200]. Similar multicenter RWS have been applied to Xuebijing Injection and Lianhua Qingwen Capsules to explore their applications in sepsis and viral pneumonia management [201]. These large datasets not only reveal adverse event signals but also conduct comparative effectiveness analyses through propensity score matching, multivariate adjustment, and target trial simulation methods, aligning TCM evaluations with international RWE standards [202].

The clinical translation of nanomedicine in TCM must also rely on RWE. The real‐world ANASTASE study (NCT05609903) by Fabi et al. evaluated atezolizumab combined with albumin‐bound PTX as a first‐line treatment for PD‐L1‐positive metastatic TNBC [203]. The study showed that the results of progression‐free survival and objective response rate were consistent with those observed in the Stage‐III IMpassion130 trial, with no reports of unexpected adverse events. These approvals have generated a large database containing clinical results, safety data, and patient‐specific response patterns of a large number of people. This large‐scale real‐world dataset provides a unique opportunity to explore the clinical effectiveness and safety of multicomponent herbal therapies in complex disease contexts. By combining these real‐world clinical data with AI‐driven pharmacokinetic and pharmacodynamic modeling, researchers can identify reliable biomarkers, refine dose‐response relationships, and more accurately simulate the in vivo behavior of nanomedicine formulations.

## 6 Conclusions

The advancement of modernized TCM relies on the synergistic integration of AI and nanotechnology, transforming experiential qualitative insights into quantifiable, mechanistic scientific understanding. AI enables multimodal analysis and intelligent interpretation of the four diagnostic methods in TCM, advancing the objectivity and standardization of clinical diagnosis. Concurrently, AI‐guided nanotechnology provides nanoscale precision in drug delivery and active targeting, establishing a closed‐loop system integrating intelligent TCM diagnosis, mechanism analysis, and therapeutic intervention—bridging macro‐level TCM syndromes with microlevel biological processes. However, challenges persist, including insufficient data standardization, limited model interpretability, inadequate nanotoxicological assessment, and insufficient clinical validation. Future research should focus on establishing large‐scale, high‐quality TCM datasets, developing interpretable AI models, and integrating nanotechnology‐based diagnostic and therapeutic platforms to achieve the intelligent, evidence‐based and personalized modern transformation of TCM.

## Author Contributions

Wenqi Yu: writing – original draft and editing and funding acquisition. Mengzhen Chen: writing – original draft and editing. Xueqi Tan: writing – original draft and editing. Xi Wei: writing – review and editing. Fan Sun: writing – review and editing. Hua Yan: writing – review and editing. Hongcai Shang: writing – review and editing. Xue Xu: writing – review and supervision and funding acquisition. All authors have read and approved the final manuscript.

## Ethics Statement

The authors have nothing to report.

## Conflicts of Interest

The authors declare no conflicts of interest.

## Data Availability

Data availability is not applicable to this article as no new data were created or analyzed in this study.
